# Metabolic-epigenetic rewiring of CCR5^hi^ monocytes sustains long-term trained immunity against lethal sepsis

**DOI:** 10.1126/sciadv.aee1634

**Published:** 2026-08-21

**Authors:** Lingqi Xu, Wenyan Hao, Yingyi Yang, Yu Wang, Yaoshuang Li, Yuan Gong, Yifang Ding, Jie Huang, Zhenjiang Bai, Qiang Shan, Rui Kang, Jiang Huai Wang, Haichao Wang, Timothy R. Billiar, Jian Wang, Daolin Tang, Huiting Zhou

**Affiliations:** ^1^ Institute of Pediatric Research, Children’s Hospital of Soochow University, Suzhou, Jiangsu, China.; ^2^ Department of Surgery, UT Southwestern Medical Center, Dallas, TX 75390, USA.; ^3^ National Key Laboratory of Immunity and Inflammation, Suzhou Institute of Systems Medicine, Chinese Academy of Medical Sciences and Peking Union Medical College, Suzhou, Jiangsu, China.; ^4^ Department of Academic Surgery, University College Cork, Cork University Hospital, Cork, Ireland.; ^5^ Laboratory of Emergency Medicine, the Feinstein Institutes for Medical Research, Manhasset, NY 11030, USA.; ^6^ Department of Surgery, University of Pittsburgh, Pittsburgh, PA 15219, USA.; ^7^ Department of Surgery, Children’s Hospital of Soochow University, Suzhou, Jiangsu, China.

## Abstract

Trained immunity enhances innate host defense by endowing monocytes with memory-like properties, yet the underlying integrated metabolic and epigenetic mechanisms remain elusive. Here, we demonstrate that coimmunization with Bacille Calmette-Guérin (BCG) and bacterial lipoprotein (BLP) induces a durable form of trained immunity that provides robust, long-term protection against polymicrobial sepsis from early life into adulthood. Single-cell RNA sequencing revealed that this effect is mediated by an expansion of CCR5^hi^ memory-like monocytes with enhanced antimicrobial capacity. Mechanistically, BCG + BLP vaccination activated the AKT–mTOR–HIF-1α axis, driving glycolytic reprogramming and lactate accumulation. Elevated lactate enhanced KAT2B-dependent histone H3K18 lactylation, an epigenetic mark directly facilitating the transcription of phagocytic and inflammatory genes. In addition, BCG + BLP stimulation of human cord blood mononuclear cells induced CCR5^hi^ monocytes that recapitulated trained immunity features. These findings identify a lactate-KAT2B-H3K18la epigenetic axis that orchestrates the long-term reprogramming of CCR5^hi^ monocytes, highlighting CCR5^hi^ monocytes as a promising therapeutic target for modulating innate immunity against lethal sepsis.

## INTRODUCTION

Sepsis, a life-threatening organ dysfunction caused by dysregulated host response to infection, represents a major global health burden (*1*, *2*). Affecting more than 50 million people annually, it contributes to approximately 11 million deaths, accounting for nearly one-fifth of global mortality (*2*–*4*). Although sepsis can affect individuals of all ages, infants, the elderly, and individuals with chronic conditions such as cancer, immunodeficiency, or kidney disease are particularly vulnerable (*2*, *4*, *5*). Despite the central role of immune dysfunction in its pathogenesis, current clinical management remains largely supportive, with few effective immunomodulatory strategies (*2*). This highlights an urgent need for prophylactic approaches that bolster innate immune resilience, particularly in high-risk populations.

Contrary to the long-held view that innate immunity lacks memory, accumulating evidence demonstrates that innate immune cells can acquire long-term functional adaptation through metabolic and epigenetic reprogramming, a process termed trained immunity (*6*–*8*). This process enables monocytes, macrophages, natural killer (NK) cells, and innate lymphoid cells to mount augmented responses upon subsequent stimulation, conferring broad-spectrum protection against pathogens (*6*, *7*, *9*–*11*). These findings suggest a potential framework for vaccine strategies that engage innate immune memory, which may provide opportunities for preventing sepsis, particularly in individuals with immature or impaired adaptive immunity.

The Bacille Calmette-Guérin (BCG) vaccine, a live-attenuated *Mycobacterium bovis* strain, is widely administered in neonates for tuberculosis prevention as the prototypical inducer of trained immunity (*12*–*16*). Beyond its mycobacterial effects, BCG reprogrammed myeloid cells to confer broad protection against unrelated infections and inflammatory diseases (*13*–*15*, *17*–*19*) and improves sepsis outcomes in neonatal models (*20*). However, these nonspecific benefits are often transient and heterogeneous across individuals, influenced by vaccine viability, administration route, and host immune status (*21*–*26*). In addition, the attenuation of BCG may diminish its capacity to elicit durable trained immunity (*22*, *27*, *28*), highlighting a need for strategies to augment and extend BCG-induced innate memory.

Bacterial lipoproteins (BLPs), abundant components of bacterial membranes and potent Toll-like receptor 2 agonists, represent promising candidates to augment BCG-induced trained immunity (*29*). BLPs reprogram innate phagocytes to enhance phagolysosomal maturation and antimicrobial responses while limiting excessive inflammation (*30*, *31*). We previously demonstrated that coadministration of BCG and BLP (BCG + BLP) in neonates enhances resistance to polymicrobial infection by amplifying antimicrobial responses (*32*); however, the precise mechanisms underlying this synergy and its long-term durability remained undefined.

Here, we demonstrated that BCG + BLP coimmunization not only enhanced the magnitude but also extended the durability of trained immunity, conferring sustained protection against sepsis from early life into adulthood. Single-cell RNA sequencing (scRNA-seq) identified a distinct population of C-C motif chemokine receptor 5 (CCR5)^hi^ memory-like monocytes specifically induced by BCG + BLP. Functional validation using adoptive transfer and myeloid-specific *Ccr5* deletion confirmed that CCR5^hi^ monocytes serve as effector cells mediating long-term protection against sepsis. Mechanistically, these cells underwent glycolytic reprogramming and lactate accumulation, which, in turn, triggered lysine acetyltransferase 2B (KAT2B)-dependent histone H3 lysine 18 (H3K18) lactylation and transcriptional activation of immune effector genes. Genetic ablation of *Kat2b* impaired BCG + BLP–induced protection in septic mice, supporting the functional importance of this metabolic-epigenetic axis. Crucially, similar features of CCR5^hi^ monocyte trained immunity were observed in human umbilical cord blood following BCG + BLP stimulation. Together, these findings define a metabolic-epigenetic circuit in CCR5^hi^ monocytes that underlies durable trained immunity against lethal sepsis, providing mechanistic insight into how combinatorial immunization strategies enhance both the magnitude and persistence of trained immunity, with potential implications for sepsis prevention.

## RESULTS

### BCG + BLP–induced trained immunity confers long-term protection against sepsis

**Fig. 1.**
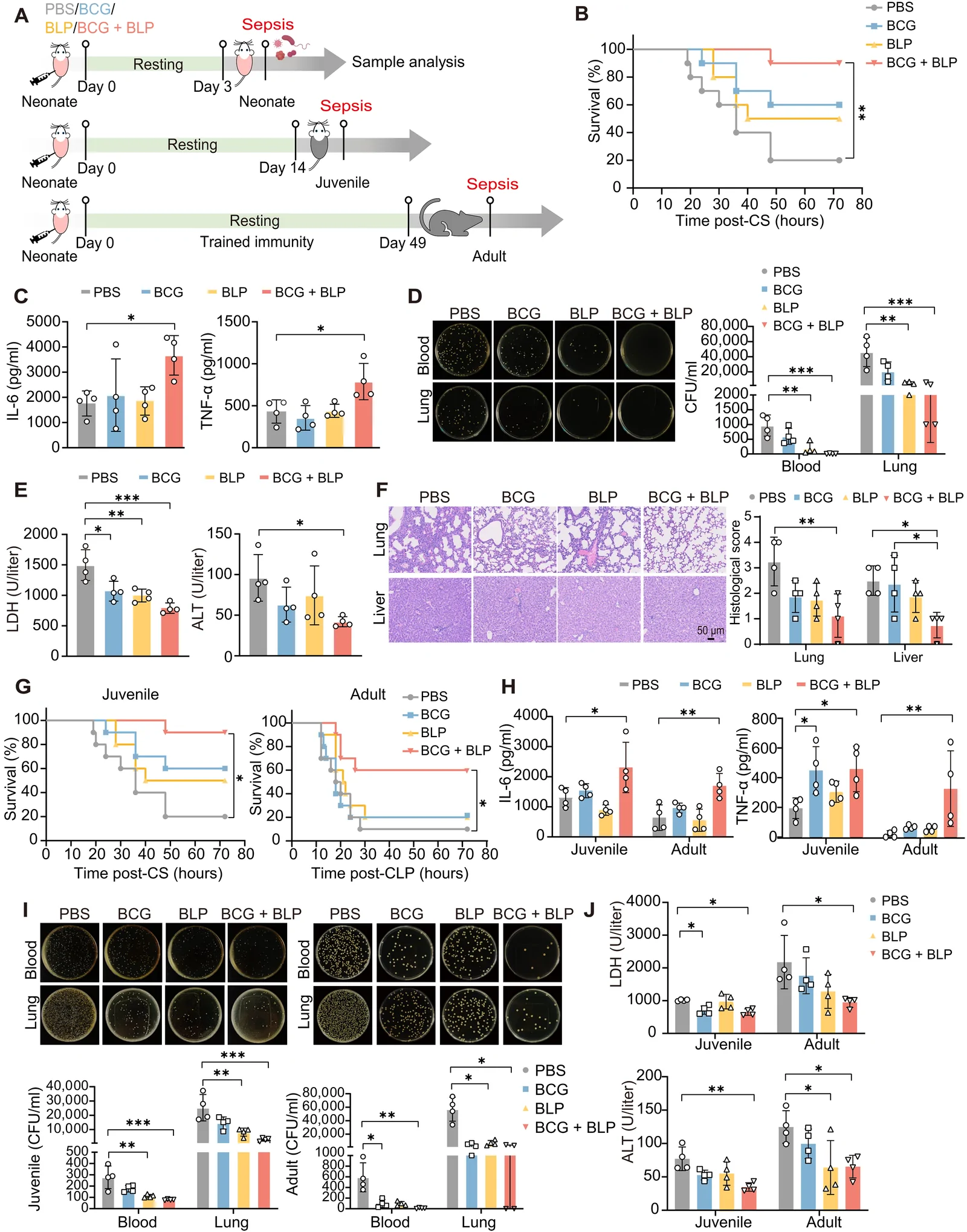
BCG + BLP–induced trained immunity confers long-term protection against sepsis. (**A**) Schematic of BCG + BLP–induced trained immunity and subsequent assays. Seven-day-old mice were treated with PBS, BCG, BLP, or BCG + BLP. Polymicrobial sepsis was induced by CS at 3 days and 2 weeks postimmunization or by cecal ligation and puncture (CLP) at 7 weeks postimmunization. (**B**) Survival after CS challenge at 3 days postimmunization (*n* = 10 mice per group). (**C**) Serum IL-6 and TNF-α levels 1 hour after CS challenge (*n* = 4). (**D**) Bacterial loads in blood and lungs 24 hours after CS challenge, with representative plates and quantification (*n* = 4). (**E**) Serum LDH and ALT levels 24 hours after CS challenge (*n* = 4). (**F**) Hematoxylin and eosin (H&E) staining of lung and liver tissues 24 hours after CS challenge. Scale bar, 50 μm. Histological score is shown (*n* = 4). (**G**) Survival after sepsis induction at 2 weeks (left) and 7 weeks (right) postimmunization (*n* = 10). (**H**) Serum IL-6 and TNF-α levels 1 hour after sepsis induction at 2 weeks and 7 weeks (*n* = 4). (**I**) Bacterial loads in blood and lungs 24 hours after sepsis induction at 2 weeks and 7 weeks postimmunization, with representative plates and quantification (*n* = 4). (**J**) Serum LDH and ALT levels 24 hours after sepsis induction at 2 weeks and 7 weeks postimmunization (*n* = 4). Data are representative of at least three independent experiments [(C) to (E) and (H) to (J)] or two independent experiments (F). Data are shown as means ± SD. Statistics: one-way analysis of variance (ANOVA) [(C) to (E) and (H) to (J)] and log-rank test [(B) and (G)]. *P < 0.05, **P < 0.01, and ***P < 0.001.

To evaluate whether coadministration of BCG and BLP enhances both the magnitude and durability of trained immunity, neonatal mice were immunized with phosphate-buffered saline (PBS), BCG, BLP, or BCG + BLP, followed by a cecal slurry (CS)–induced polymicrobial sepsis 3 days later (Fig. 1A). Mice receiving BCG + BLP exhibited notably improved survival (90%) compared to PBS controls (20%) and superior protection over BCG or BLP alone (Fig. 1B). This protection correlated with a rapid, early inflammatory response, marked by elevated interleukin-6 (IL-6) and tumor necrosis factor–α (TNF-α) within 1 hour of sepsis induction (Fig. 1C). BCG + BLP training also enhanced pathogen clearance, reducing bacterial loads in the blood and major organs (lungs, spleen, and liver) (Fig. 1D and fig. S1A). In addition, serum tissue injury markers, including lactate dehydrogenase (LDH) and alanine aminotransferase (ALT), were lowest in the BCG + BLP group (Fig. 1E), and histopathological analysis confirmed reduced tissue damage and inflammatory cell infiltration in the lung and liver (Fig. 1F).

BCG + BLP also conferred a similar protective phenotype in 6- to 8-week-old mice challenged with *Escherichia coli* [4 × 10^6^ colony-forming units (CFU)] or cecal ligation and puncture (CLP), consistent with improved survival across diverse sepsis etiologies (fig. S1, B and C). This protection extended to aged mice, with BCG + BLP–immunized animals showing 80% surviving in CLP-induced sepsis, higher than PBS (25%), BCG (40%), or BLP (50%) (fig. S1D).

To determine whether this protection persists beyond the neonatal stage, we next challenged mice at 2 or 7 weeks postimmunization, representing juvenile and adult stages, respectively (*33*–*36*). BCG + BLP–treated mice maintained high survival rates at both time points, outperforming all other groups (Fig. 1G). This long-term protection correlated with sustained elevations in early inflammatory cytokines (IL-6 and TNF-α) (Fig. 1H), improved bacterial clearance (Fig. 1I and fig. S1, E and F), and reduced tissue damage, as reflected by decreased serum LDH and ALT levels (Fig. 1J). Collectively, these findings suggest that BLP functions as an immunomodulatory adjuvant that can strengthen both the magnitude and durability of BCG-induced trained immunity.

### Expansion of CCR5^hi^ monocytes characterizes BCG + BLP–induced trained immunity

**Fig. 2.**
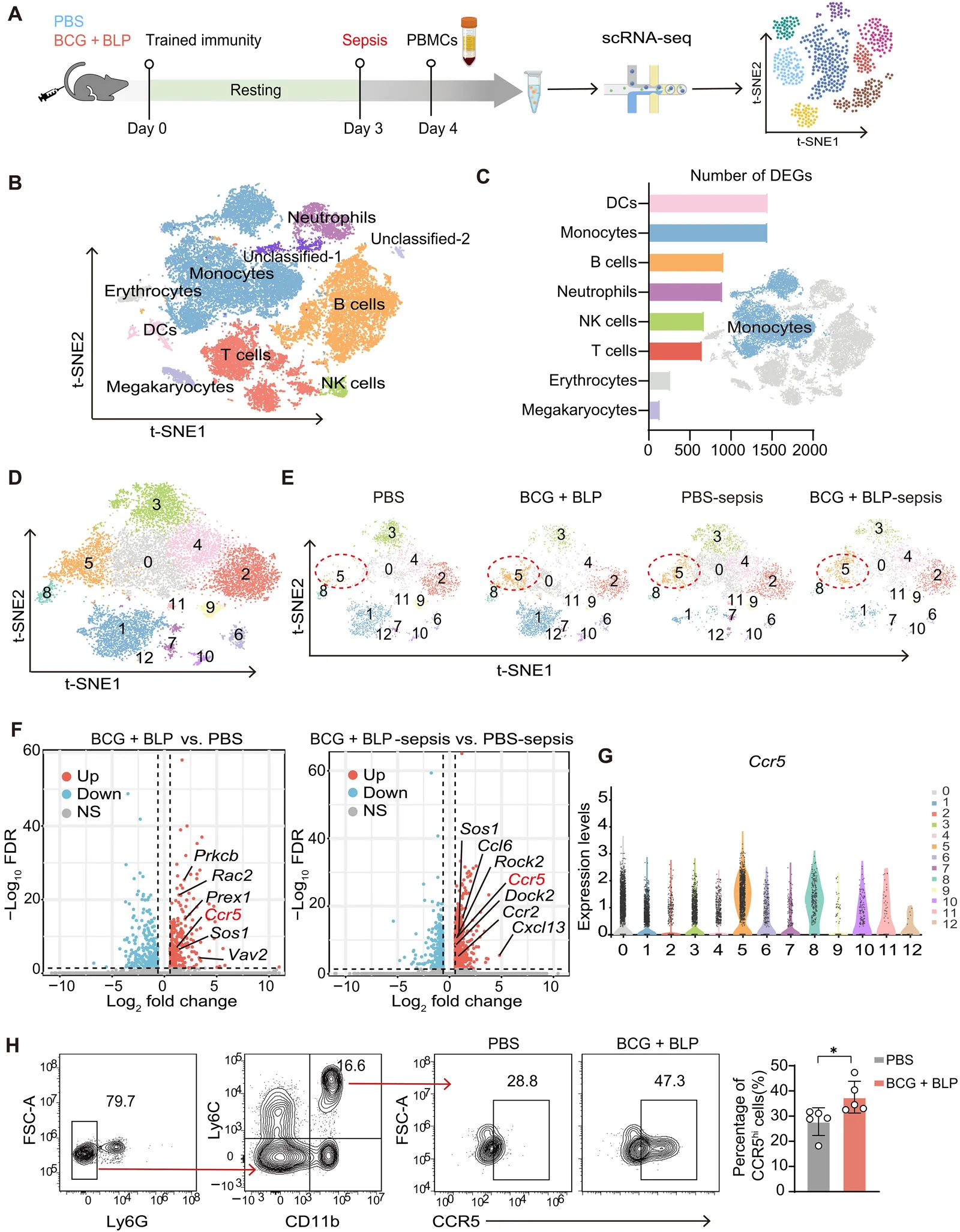
Expansion of CCR5^hi^ monocytes characterizes BCG + BLP–induced trained immunity. (**A**) Schematic of the scRNA-seq pipeline for PBMCs isolated from 7-day-old mice treated with PBS or BCG + BLP, with or without polymicrobial sepsis induction. (**B**) t-distributed stochastic neighbor embedding (t-SNE) plots of 31,132 immune cells allocated into 10 clusters from PBMCs of mice in PBS, BCG + BLP, PBS-sepsis, and BCG + BLP-sepsis groups. PBMCs from six to eight mice were pooled as one sample. (**C**) Bar plots showing the distribution of DEGs across immune cell types in the BCG + BLP group compared with PBS controls. (**D**) t-SNE plots of 12,588 monocytes from (C) allocated to the 13 clusters. (**E**) t-SNE plots of the monocytes from the indicated groups, annotated by cluster as in (D). (**F**) Volcano plots showing fold changes in gene expression (*x* axis, log_2_ scale) and *P* value significance (*y* axis, −log_10_ scale) in Mon5 subset: BCG + BLP versus PBS (left) and BCG + BLP-sepsis versus PBS-sepsis (right). Selected significant genes are labeled. Two-sided *P* values were determined using the Mann-Whitney *U* test. (**G**) Violin plots showing *Ccr5* expression across monocyte clusters corresponding to (D). (**H**) Flow cytometric analysis of CD11b^+^Ly6G^−^Ly6C^+^CCR5^hi^ monocytes in the indicated groups, with quantification (*n* = 5 mice per group). Data are representative of at least three independent experiments (H) and are shown as means ± SD. Statistics: two-tailed unpaired Student’s *t* test (H). *P < 0.05. DCs, dendritic cells; FDR, false discovery rate; NS, not significant.

To identify the effector cell populations responsible for BCG + BLP–induced trained immunity, we performed scRNA-seq on peripheral blood mononuclear cells (PBMCs) from neonatal mice treated with PBS or BCG + BLP (Fig. 2A). After quality control, a total of 31,132 cells were analyzed, with an average of 8425 unique molecular identifier (UMI) counts and 2683 detected genes per cell (fig. S2A). Unbiased clustering analysis revealed distinct immune cell populations, including monocytes (*Csf1r* and *Lyz2*), B cells (*Cd79a* and *Ms4a1*), T cells (*Cd3d*, *Cd3e*, and *Cd3g*), neutrophils (*Cd14*, *Ngp*, and *S100a8*), dendritic cells (*Cd74*), megakaryocytes (*Ppbp*), erythrocytes (*Hba-a1* and *Hba-a2*), and NK cells (*Nkg7* and *Klrb1c*) (Fig. 2B and fig. S2, B and C). Among these, monocytes from the BCG + BLP–treated group exhibited a substantial number of differentially expressed genes (DEGs) compared to PBS-treated controls (Fig. 2C), indicating that monocytes are a dynamically reprogrammed population in response to BCG + BLP–induced trained immunity.

To further dissect the heterogeneity of the monocyte compartment, we performed unsupervised clustering, which identified 13 monocyte subsets (Mon0 to Mon12) (Fig. 2D). Among these, Mon5 cells were expanded in BCG + BLP–trained mice compared to PBS controls, both under steady-state conditions and following CS-induced sepsis (Fig. 2E and fig. S2D).

Functional analysis of Mon5 cells using volcano plots revealed marked up-regulation of genes in the BCG + BLP group relative to controls, independent of septic challenge (Fig. 2F). The chemokine receptor CCR5, which mediates immune cell recruitment and activation (*37*–*39*), was enriched in Mon5 cells (Fig. 2G). Flow cytometry confirmed CCR5 as a defining surface marker of this subset, showing an increased frequency of CD11b^+^Ly6G^−^Ly6C^+^CCR5^hi^ monocytes in BCG + BLP–trained mice compared to controls at day 3 in peripheral blood (Fig. 2H). CCR5^hi^ monocytes persisted at elevated levels in peripheral blood, spleen, and bone marrow at days 14 and 28 after training, indicating their long-term maintenance following BCG + BLP immunization (fig. S2, E to G). Together, these results identify CCR5^hi^ Mon5 cells as a distinct monocyte subpopulation expanded by BCG + BLP immunization.

### CCR5^hi^ monocytes as effector cells in BCG + BLP–induced trained immunity

**Fig. 3.**
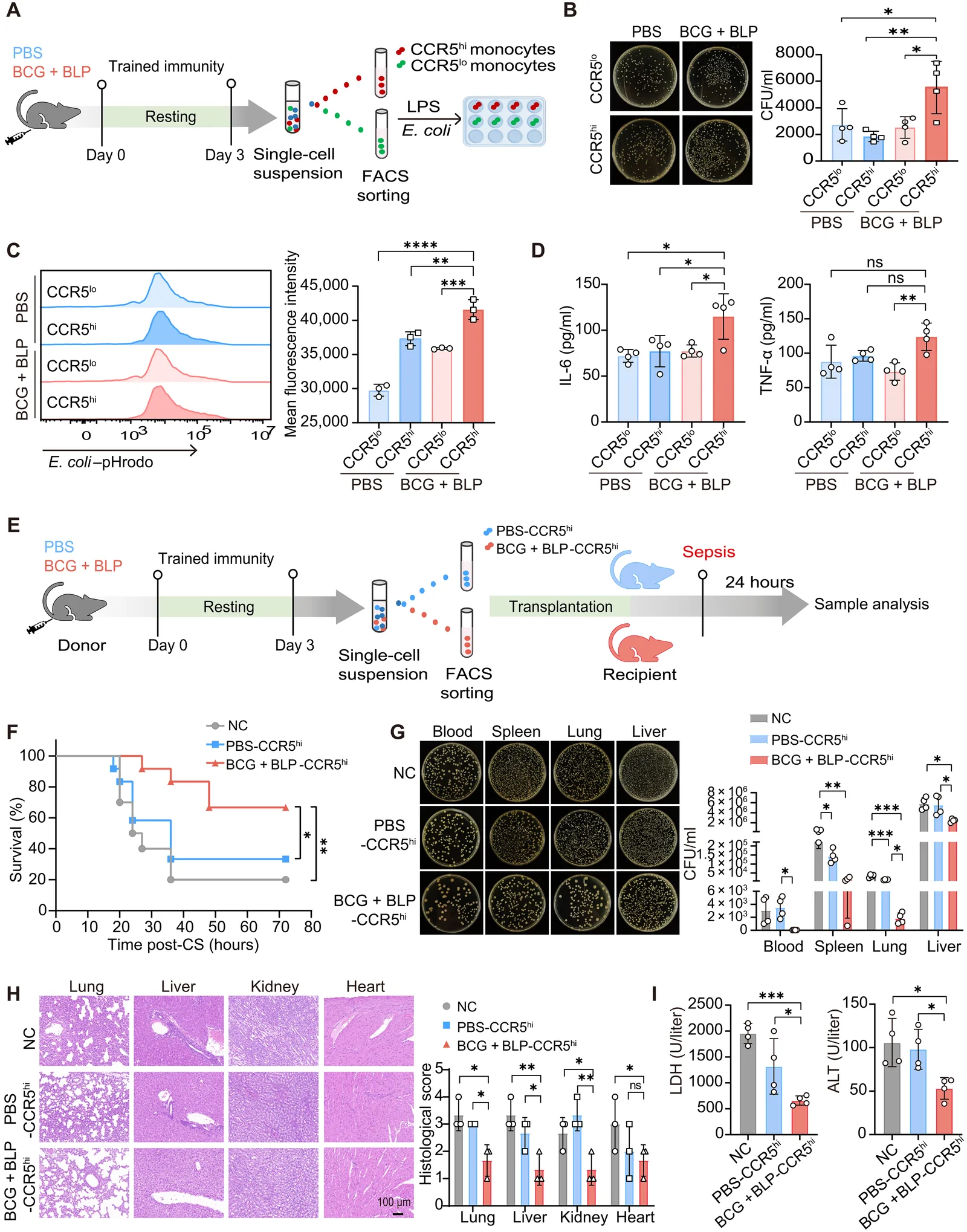
CCR5^hi^ monocytes as key effector cells in BCG + BLP–induced trained immunity. (**A**) Schematic illustration for ex vivo validation of CCR5^hi^ Mon5 cells. Seven-day-old mice were treated with PBS or BCG + BLP. After a 3-day resting period, bone marrow–derived monocytes were isolated as PBS-CCR5^lo^, PBS-CCR5^hi^, BCG + BLP-CCR5^lo^, and BCG + BLP-CCR5^hi^ and stimulated with *E. coli* or LPS. (**B** and **C**) Phagocytosis of sorted monocytes assessed by CFU assay (B) and flow cytometry (C) after incubation with fluorescent *E. coli* for 30 min (*n* = 3 to 4). (**D**) IL-6 and TNF-α levels in culture supernatants 12 hours after LPS stimulation (*n* = 4). (**E**) Schematic illustration of adoptive transfer. CCR5^hi^ monocytes sorted from PBS- or BCG + BLP–treated donor mice were intravenously transferred into naïve recipient mice. PBS injection served as a negative control (NC). Recipients were challenged with CS 2 hours later. (**F**) Survival of recipient mice after CS challenge (*n* = 10). (**G**) Bacterial loads in blood, spleen, lung, and liver 24 hours after CS challenge, with representative plates and quantification (*n* = 4). (**H**) H&E staining of lung, liver, kidney, and heart tissues 24 hours after CS challenge. Scale bar, 100 μm. Histological score is shown (*n* = 3). (**I**) Serum LDH and ALT levels 24 hours after CS challenge (*n* = 4). Data are representative of at least three independent experiments [(B) to (D) and (G) and (I)] or two independent experiments (H). Data are shown as means ± SD. Statistics: one-way ANOVA [(B) to (D) and (G) and (I)] and log-rank test (F). *P < 0.05, **P < 0.01, ***P < 0.001, and ****P < 0.0001. FACS, fluorescence-activated cell sorting; ns, not significant.

To determine whether CCR5^hi^ Mon5 cells develop innate immune memory, neonatal mice were intraperitoneally injected with either PBS or BCG + BLP. After a 3-day resting period, bone marrow–derived monocytes were isolated by flow cytometry based on CD45^+^CD11b^+^Ly6G^−^Ly6C^+^CCR5^lo^ or CCR5^hi^ expression. These sorted cells (PBS-CCR5^lo^, PBS-CCR5^hi^, BCG + BLP-CCR5^lo^, and BCG + BLP-CCR5^hi^) were subsequently exposed ex vivo to *E. coli* or lipopolysaccharide (LPS) to evaluate their functional responses (Fig. 3A; gating strategy shown in fig. S3A). Phagocytic capacity, assessed by CFU assays after a 30-min *E. coli* incubation, was highest in BCG + BLP-CCR5^hi^ monocytes compared to all other groups (Fig. 3B). These findings were supported by a phagosome acidification assay using pHrodo-labeled *E. coli* bioparticles, which further confirmed enhanced phagocytic activity in the BCG + BLP-CCR5^hi^ group (Fig. 3C). In addition, cytokine measurements 12 hours post-LPS stimulation showed that BCG + BLP-CCR5^hi^ monocytes produced the highest levels of IL-6 and TNF-α (Fig. 3D). Thus, BCG + BLP training imparts memory-like properties to CCR5^hi^ monocytes, enhancing both their phagocytic and cytokine-producing capacities upon challenge.

To further validate the role of CCR5 signaling, we administered the CCR5 antagonist maraviroc (MVC). Seven-day-old mice were intraperitoneally injected with PBS or BCG + BLP, with or without MVC. After a 3-day resting period, the frequency of CCR5^hi^ monocytes was assessed (fig. S3B). Consistent with the above findings, BCG + BLP increased the proportion of CCR5^hi^ monocytes compared to PBS controls. Pharmacological inhibition of CCR5 reduced the expansion of this subset, suggesting that CCR5 signaling contributes to the generation of CCR5^hi^ monocytes (fig. S3, C and D). Furthermore, BCG + BLP–induced protection against sepsis, as reflected by reduced bacterial burdens in blood and lung, was impaired upon CCR5 inhibition, with increased bacterial loads observed in MVC-treated mice (fig. S3E). These results demonstrate that CCR5 signaling plays a functional role in trained immunity.

To assess the functional relevance of CCR5^hi^ monocytes in mediating protection against sepsis, we conducted adoptive transfer experiments. Donor mice were intraperitoneally injected with either PBS (control) or BCG + BLP. After a 3-day resting period, bone marrow CCR5^hi^ monocytes were sorted by flow cytometry and intravenously transferred into naïve recipient mice. An additional group receiving PBS alone served as a negative control (NC). Two hours after cell transfer, all recipient mice were subjected to CS-induced polymicrobial sepsis (Fig. 3E). Mice receiving CCR5^hi^ monocytes from BCG + BLP–trained donors exhibited notably higher survival than NC mice and those receiving CCR5^hi^ monocytes from PBS-treated donors (Fig. 3F). This protective effect was accompanied by reduced bacterial loads in the blood, spleen, lungs, and liver (Fig. 3G). Histological examination of lung, liver, kidney, and heart tissues further confirmed reduced tissue injury and inflammatory infiltration in the BCG + BLP-CCR5^hi^ recipient group (Fig. 3H). Consistently, these mice also exhibited lower serum levels of tissue damage markers, including LDH and ALT (Fig. 3I). Together, these findings identify CCR5^hi^ monocytes as key effector cells in BCG + BLP–induced trained immunity, acquiring memory-like functional properties that confer protection against sepsis upon adoptive transfer.

### CCR5^hi^ monocytes mediate long-term trained immunity against sepsis

**Fig. 4.**
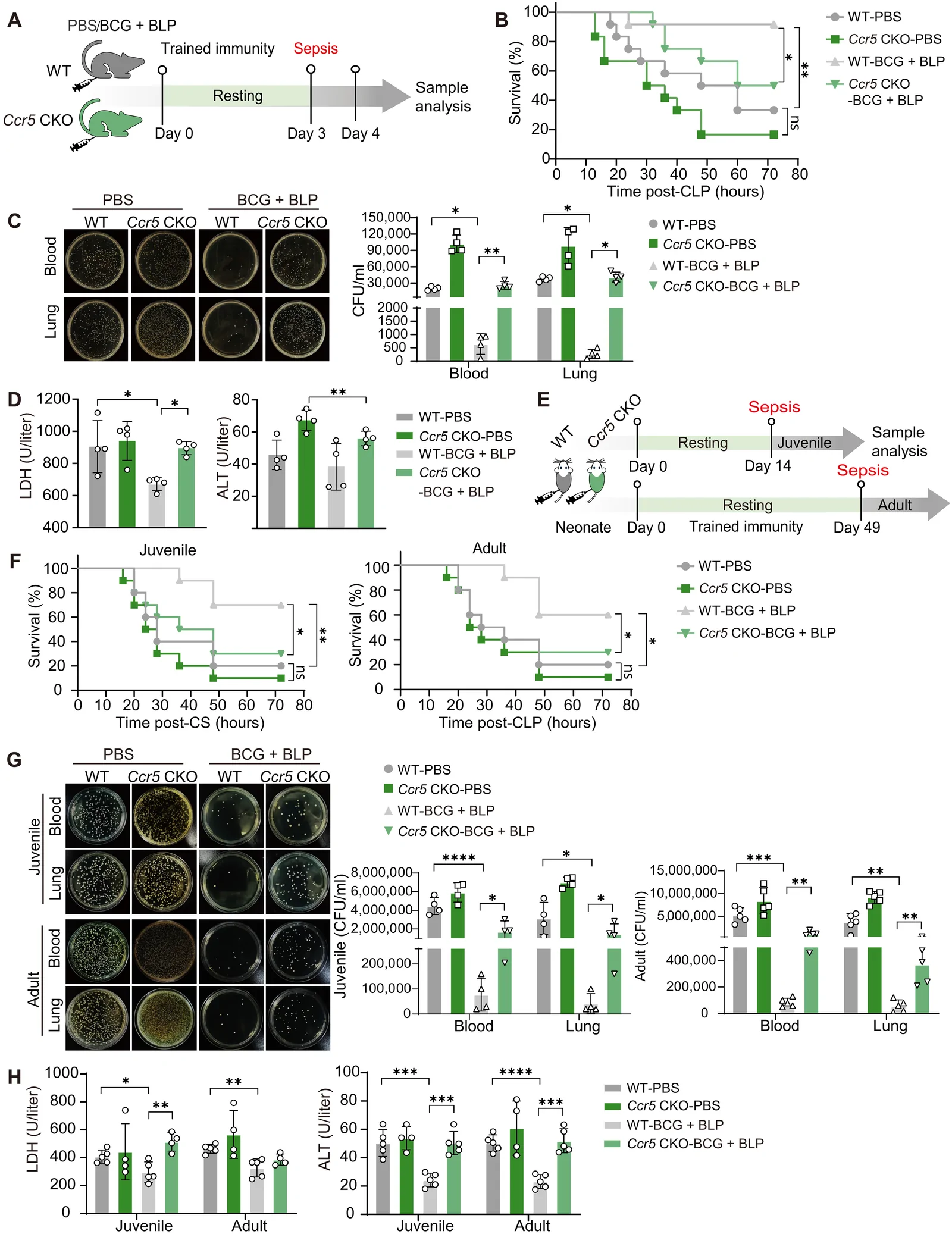
CCR5^hi^ monocytes mediate long-term trained immunity against sepsis. (**A**) Schematic diagram of in vivo functional assessment. Six- to 8-week-old WT and myeloid-specific CCR5-deficient (*Ccr5* CKO) mice were immunized with PBS or BCG + BLP. After a 3-day resting period, polymicrobial sepsis was induced, and survival, tissue injury, and bacterial burden were assessed. (**B**) Survival of WT and *Ccr5* CKO mice after sepsis induction (*n* = 10). (**C**) Bacterial loads in blood and lung 24 hours after sepsis induction, with representative plates and quantification (*n* = 4). (**D**) Serum LDH and ALT levels 24 hours after sepsis induction (*n* = 4). (**E**) Schematic diagram of in vivo functional assessment. Seven-day-old WT and *Ccr5* CKO mice were immunized with PBS or BCG + BLP. Polymicrobial sepsis was induced by CS injection at 2 weeks postimmunization or by CLP at 7 weeks postimmunization. (**F**) Survival of WT and *Ccr5* CKO mice after sepsis induction (*n* = 10). (**G**) Bacterial loads in blood and lung 24 hours after sepsis induction, with representative plates and quantification (*n* = 4 to 5). (**H**) Serum LDH and ALT levels 24 hours after sepsis induction (*n* = 4 to 5). Data are representative of at least three independent experiments [(C), (D), (G), and (H)] and are shown as means ± SD. Statistics: one-way ANOVA [(C, (D), (G), and (H)] and log-rank test [(B) and (F)]. *P < 0.05, **P < 0.01, ***P < 0.001, and ****P < 0.0001.

To assess the functional importance of CCR5^hi^ monocytes in vivo, 6- to 8-week-old wild-type (WT) and myeloid-specific *Ccr5*-deficient (*Ccr5*^fl/fl^ *Lyz2*^cre^; hereafter referred to as *Ccr5* conditional knockout [CKO]) mice were immunized with PBS or BCG + BLP. After a 3-day resting period, mice were subjected to CS-induced polymicrobial sepsis, and survival, tissue injury, and bacterial burden were evaluated (Fig. 4A). As expected, BCG + BLP–immunized WT mice displayed improved survival compared to PBS controls. However, this survival benefit was reduced in *Ccr5* CKO mice, with survival rates dropping from 90% in WT to 50% in knockout mice (Fig. 4B). Consistently, BCG + BLP–treated *Ccr5* CKO mice exhibited elevated bacterial loads in blood and multiple organs, including lung, spleen, and liver (Fig. 4C and fig. S4A), along with increased serum levels of tissue damage markers (LDH and ALT) (Fig. 4D).

To further investigate the durability of protection conferred by CCR5^hi^ monocytes in vivo, neonatal WT and *Ccr5* CKO mice were similarly immunized with PBS or BCG + BLP and challenged with sepsis at either 2 or 7 weeks postimmunization (Fig. 4E). At both time points, BCG + BLP–immunized WT mice maintained improved survival, whereas *Ccr5* CKO mice failed to sustain this benefit, with survival declining from 70 to 30% in juvenile mice and from 60 to 30% in adult mice, respectively (Fig. 4F). Correspondingly, bacterial burden and tissue injury markers remained increased in *Ccr5* CKO mice compared to WT mice at both time points (Fig. 4, G and H). Together, these results demonstrate that CCR5^hi^ monocytes trained by BCG + BLP acquire memory-like properties that enhance antimicrobial and inflammatory responses, thereby mediating long-term protection against sepsis through trained immunity.

### Glycolytic reprogramming characterizes CCR5^hi^ monocytes in trained immunity

**Fig. 5.**
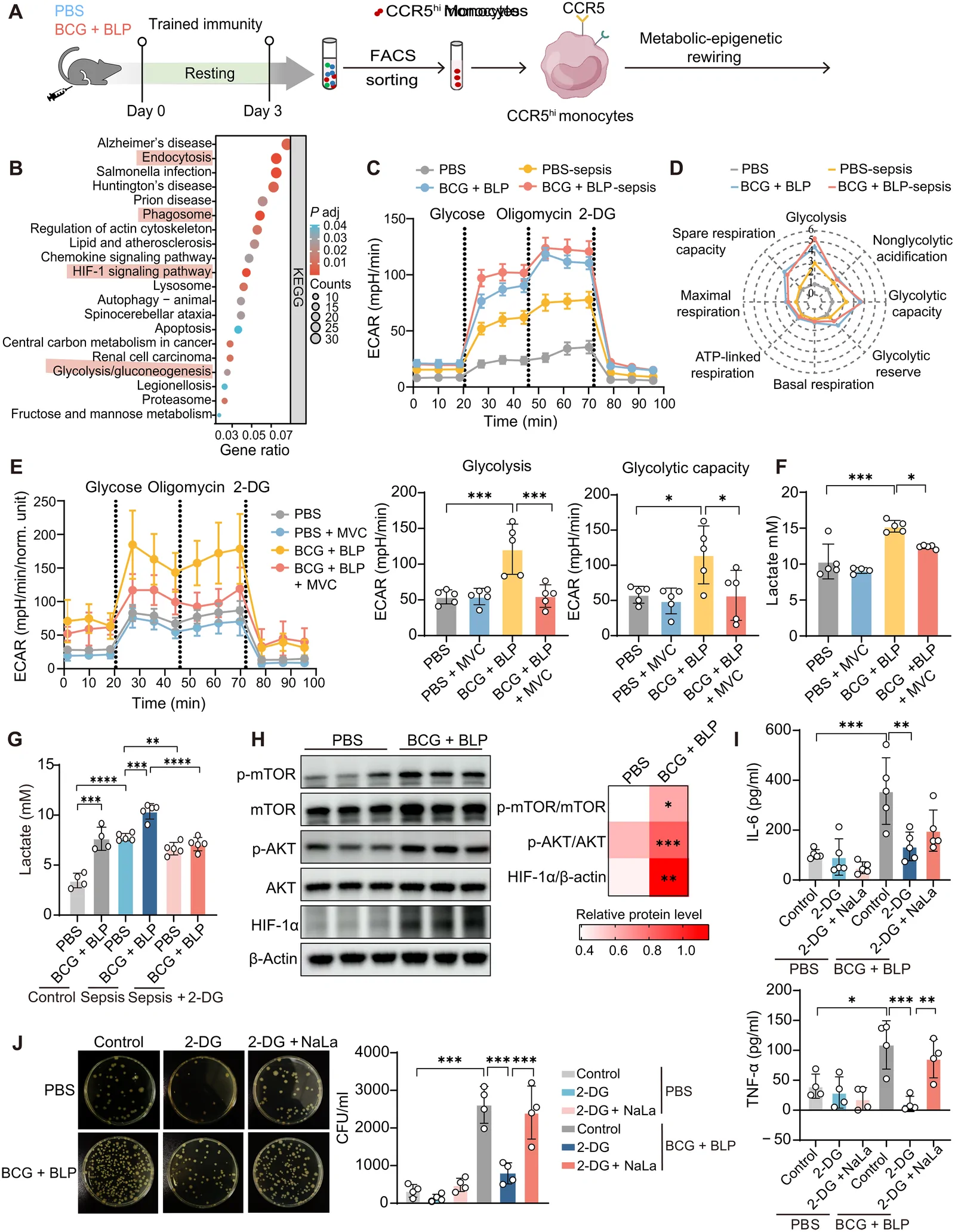
Glycolytic reprogramming characterizes CCR5^hi^ monocytes in trained immunity. (**A**) Schematic illustration showing the validation of metabolic-epigenetic rewiring in CCR5^hi^ monocytes. Seven-day-old mice were treated with PBS or BCG + BLP. After a 3-day resting period, bone marrow–derived CCR5^hi^ monocytes were sorted for metabolic and epigenetic analyses. (**B**) Heatmap of DEGs between PBS and BCG + BLP groups, with KEGG enrichment of DEGs in CCR5^hi^ Mon5 cells by scRNA-seq. (**C** and **D**) CCR5^hi^ monocytes were stimulated ex vivo with LPS for 12 hours, followed by Seahorse analysis of glycolysis (C) and radar plot analysis of glycolysis and mitochondrial OXPHOS (D). (**E** and **F**) Seven-day-old mice were treated with PBS or BCG + BLP, with or without the CCR5 antagonist MVC. Glycolysis was assessed by Seahorse analysis (E), and lactate was measured in sorted CCR5^hi^ monocytes (F) (*n* = 4 to 5). (**G**) Lactate levels in CCR5^hi^ monocyte after LPS stimulation with or without 2-DG (*n* = 4 to 5). (**H**) Western blot analysis of AKT-mTOR-HIF signaling proteins in CCR5^hi^ monocytes, with densitometry (*n* = 5). (**I** and **J**) Seven-day-old mice were treated with PBS or BCG + BLP, with or without 2-DG and/or sodium lactate (NaLa). Sorted CCR5^hi^ monocytes were stimulated ex vivo with LPS or *E. coli*. IL-6 and TNF-α levels were measured 12 hours after LPS stimulation (I), and phagocytosis was assessed by CFU assay after *E. coli* incubation (J) (*n* = 4 to 5). ECAR, extracellular acidification rate; OCR, oxygen consumption rate; 2-DG, 2-deoxy-d-glucose. Data are representative of at least three independent experiments and are shown as means ± SD. Statistics: one-way ANOVA. *P < 0.05, **P < 0.01, ***P < 0.001, and ****P < 0.0001. ATP, adenosine triphosphate; p-mTOR, phosphorylated mTOR; p-AKT, phosphorylated AKT.

To investigate the functional properties of CCR5^hi^ memory-like monocytes induced by BCG + BLP, we sorted these cells and performed an integrated analysis of their metabolic-epigenetic rewiring (Fig. 5A). Kyoto Encyclopedia of Genes and Genomes (KEGG) pathway enrichment analysis of 715 up-regulated DEGs in CCR5^hi^ Mon5 cells, in addition to previously characterized enrichment in antibacterial innate immune pathways such as endocytosis and phagosome, showed enrichment in pathways related to aerobic glycolysis, including the hypoxia-inducible factor 1-α (HIF-1α) signaling pathway and glycolysis/gluconeogenesis (Fig. 5B), suggesting metabolic reprogramming.

To characterize the metabolic phenotype of CCR5^hi^ monocytes, we measured aerobic glycolysis and oxidative phosphorylation (OXPHOS) using Seahorse XF metabolic analyzers. Regardless of secondary septic challenge, CCR5^hi^ monocytes from BCG + BLP–trained mice exhibited elevated extracellular acidification rates (ECARs), indicating increased glycolytic activity compared to PBS controls (Fig. 5C and fig. S5A). Oxygen consumption rates (OCRs) were also enhanced, reflecting increased OXPHOS activity (fig. S5, B and C). However, the increase in glycolytic parameters—including glycolysis, glycolytic capacity, and glycolytic reserve—was more pronounced than the corresponding increase in OXPHOS metrics (Fig. 5D), indicating a preferential shift toward glycolysis. CCR5 inhibition by MVC attenuated BCG + BLP–induced ECAR elevation (Fig. 5E). Consistently, intracellular lactate levels, the terminal product of glycolysis, were increased in CCR5^hi^ monocytes following BCG + BLP training, independent of septic challenge (Fig. 5, F and G). This lactate accumulation was abolished by MVC treatment and was also reduced by pharmacological inhibition of glycolysis using 2-deoxy-d-glucose (2-DG) (Fig. 5, F and G).

Given the central role of the AKT–mechanistic target of rapamycin (mTOR)–HIF-1α axis in glycolytic reprogramming during trained immunity (*40*), we next assessed the activation status of these signaling components. Western blot analysis revealed elevated levels of phosphorylated AKT, phosphorylated mTOR, and HIF-1α in CCR5^hi^ monocytes from BCG + BLP–trained mice compared to controls (Fig. 5H).

To further establish the functional relevance of glycolytic metabolism, we performed metabolic intervention experiments. Neonatal mice were intraperitoneally injected with PBS or BCG + BLP, with or without 2-DG and/or sodium lactate (NaLa) supplementation. As expected, BCG + BLP–trained CCR5^hi^ monocytes exhibited enhanced production of IL-6 and TNF-α upon LPS stimulation, together with increased phagocytic capacity as assessed by CFU-based assays following *E. coli* challenge. Both cytokine production and phagocytic activity were reduced by 2-DG treatment and were partially restored by exogenous lactate supplementation (Fig. 5, I and J). Consistent results were obtained following treatment with the LDH inhibitor sodium oxamate (SO) (fig. S5, D and E), further supporting lactate as a key downstream metabolic effector of BCG + BLP–induced glycolytic reprogramming.

### Lactate-driven H3K18 lactylation programs immune memory in CCR5^hi^ monocytes

Metabolic reprogramming, a hallmark of trained immunity, not only supplies energy but also generates metabolites that directly regulate epigenetic remodeling through histone modifications (*7*, *41*). Lactate was recently identified as a precursor for histone lactylation, a lysine modification that regulates gene transcription (*42*–*44*). Given the elevated glycolytic activity and lactate accumulation observed in CCR5^hi^ monocytes following BCG + BLP training, we investigated whether this metabolic shift promotes histone lactylation.

**Fig. 6.**
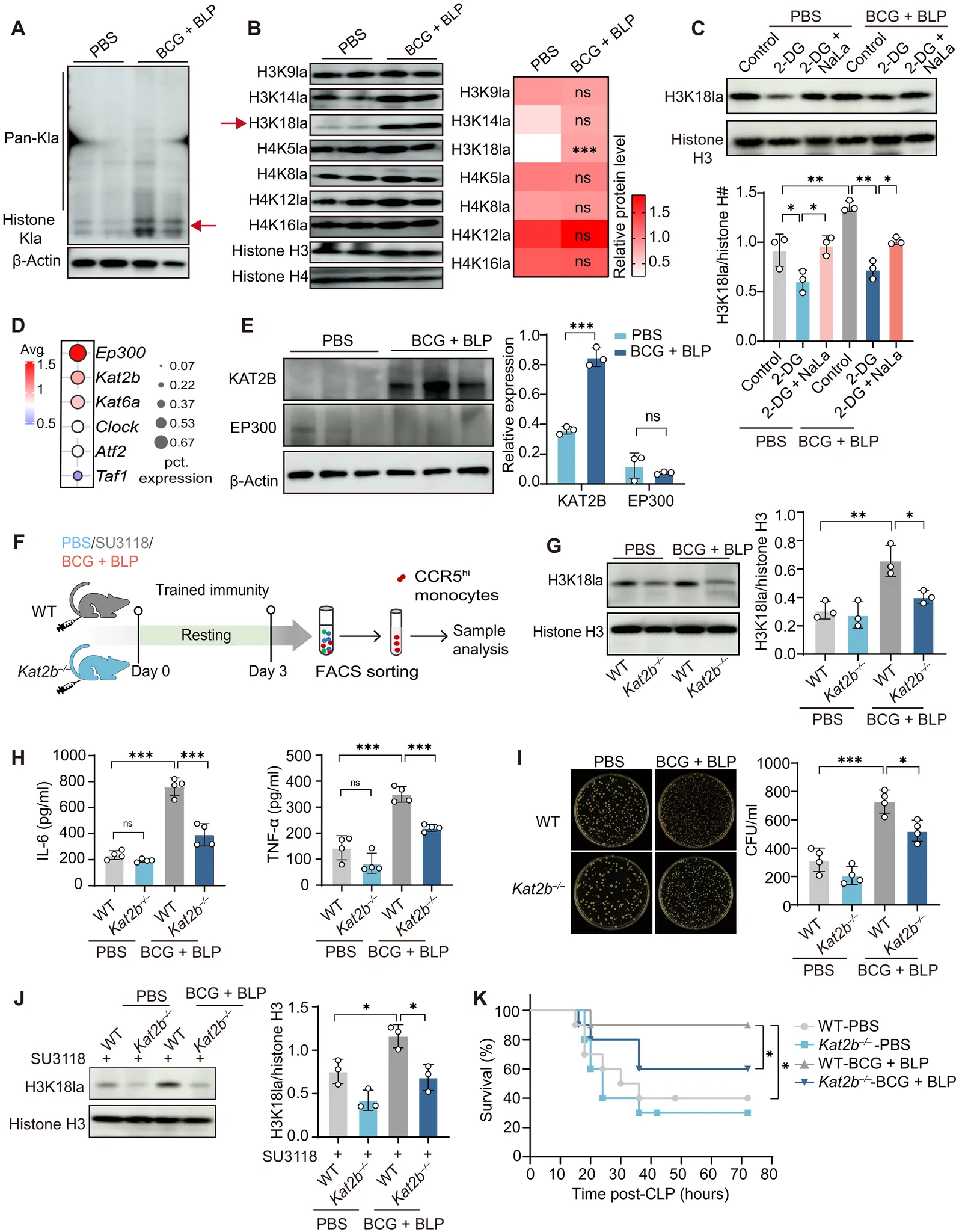
Lactate-driven H3K18 lactylation programs immune memory in CCR5^hi^ monocytes. [(A), (B), and (E)] Seven-day-old mice were treated with PBS or BCG + BLP. After a 3-day resting period, bone marrow–derived CCR5^hi^ monocytes were isolated for analyses. (**A**) Western blot analysis of global lactylated lysine (Pan-Kla). The lower molecular weight region corresponding to histone is indicated by the arrow. Actin served as loading control. (**B**) Western blot analysis of site-specific histone lactylation. H3 or H4 histone served as loading control (*n* = 3). (**C**) Western blot analysis of histone lactylation in the indicated groups, with H3 as loading control (*n* = 3). (**D**) Heatmap showing acetyltransferase expression in CCR5^hi^ Mon5 cells based on scRNA-seq data. (**E**) Western blot analysis of KAT2B and EP300 expression, with actin as loading control (*n* = 3). (**F**) Schematic diagram of functional assessment. Adult WT and *Kat2b*-deficient (*Kat2b^−/−^*) mice aged 6 to 8 weeks were immunized with PBS or BCG + BLP, with or without the MCT1/4 inhibitor syrosingopine (SU3118). CCR5^hi^ monocytes were isolated after 3 days. (**G**) Western blot analysis of H3K18la in CCR5^hi^ monocytes from WT or *Kat2b^−/−^* mice (*n* = 3). (**H**) IL-6 and TNF-α levels in supernatants 12 hours after LPS stimulation (*n* = 4). (**I**) Phagocytosis of CCR5^hi^ monocytes from WT or *Kat2b^−/−^* mice assessed by CFU assay after *E. coli* incubation (*n* = 4). (**J**) Western blot analysis of H3K18la in the indicated groups (*n* = 3). (**K**) Survival of WT and *Kat2b^−/−^* mice immunized with PBS or BCG + BLP following sepsis induction (*n* = 10). Data are representative of at least three independent experiments and are shown as means ± SD. Statistics: one-way ANOVA [(B), (C), (E), and (H) to (J)] and log-rank test (K). *P < 0.05, **P < 0.01, and ***P < 0.001. pct., percent expression.

To assess this, CCR5^hi^ monocytes were sorted from PBS- and BCG + BLP–treated mice, and total histone lysine lactylation (Kla) was evaluated by Western blot. Compared to PBS controls, BCG + BLP–trained CCR5^hi^ monocytes exhibited increased global lysine lactylation (Fig. 6A). Further analysis of specific histone sites revealed a marked enrichment of histone H3 lysine 18 lactylation (H3K18la) in the BCG + BLP group (Fig. 6B). To determine whether glycolysis is required for this epigenetic modification, we treated mice with the glycolytic inhibitor 2-DG and/or NaLa supplementation. 2-DG treatment attenuated H3K18la levels in CCR5^hi^ monocytes from both PBS- and BCG + BLP–treated groups, whereas supplementation with exogenous lactate partially restored H3K18la levels (Fig. 6C). Consistently, treatment with either the LDH inhibitor SO or the CCR5 antagonist MVC similarly attenuated BCG + BLP–induced H3K18la accumulation (fig. S6, A and B), confirming that glycolytic reprogramming drives H3K18la enrichment during BCG + BLP–induced trained immunity.

Histone lactylation is catalyzed by acetyltransferases that use lactyl–coenzyme A as a donor. Enzymes such as P300 (as known EP300) (*45*) and KAT2A (*46*) have been implicated in this process. To identify the key lactyltransferase responsible for H3K18la in BCG + BLP–trained CCR5^hi^ monocytes, we analyzed acetyltransferase expression in CCR5^hi^ Mon5 cells using scRNA-seq. Among several up-regulated enzymes, P300 and KAT2B showed the most pronounced increases (Fig. 6D). Western blot analysis, however, revealed a marked elevation of KAT2B protein but not P300 (Fig. 6E). Exogenous lactate supplementation further enhanced KAT2B expression in BCG + BLP–trained CCR5^hi^ monocytes, whereas LDH inhibition by SO reduced KAT2B protein levels (fig. S6C), suggesting that lactate signaling promotes KAT2B induction.

To validate the functional role of KAT2B, we induced trained immunity in 6- to 8-week-old WT and *Kat2b*-deficient (*Kat2b^−/−^*) mice via BCG + BLP administration (Fig. 6F). CCR5^hi^ monocytes were isolated 3 days postimmunization and analyzed for H3K18la levels. *Kat2b* deficiency impaired the induction of H3K18la in CCR5^hi^ monocytes (Fig. 6G). Moreover, CCR5^hi^ monocytes from BCG + BLP–treated *Kat2b^−/−^* mice exhibited reduced cytokine production and phagocytic activity (Fig. 6, H and I), indicating a loss of trained immunity–associated functional enhancements. Similar effects were observed following inhibition of intracellular lactate transport using the dual monocarboxylate transporters 1 and 4 (MCT1/4) inhibitor syrosingopine (SU3118). Under these conditions, *Kat2b* deficiency still impaired H3K18la induction, cytokine production, and phagocytic activity in CCR5^hi^ monocytes (Fig. 6J and fig. S6, D and E).

To corroborate these findings in vivo, 6- to 8-week-old WT and *Kat2b^−/−^* mice were immunized with PBS or BCG + BLP and challenged with sepsis 3 days postimmunization. As expected, BCG + BLP–immunized WT mice showed improved survival compared to PBS controls, reaching up to 90% survival. However, this protective effect was reduced in *Kat2b^−/−^* mice, with survival rates decreasing to 60% (Fig. 6K). Consistently, BCG + BLP–treated *Kat2b^−/−^* mice exhibited increased serum levels of tissue damage markers (LDH and ALT) (fig. S6F). Collectively, these results demonstrate that BCG + BLP–induced trained immunity in CCR5^hi^ monocytes is mediated through KAT2B-dependent H3K18 lactylation.

### Epigenetic activation of CCR5^hi^ monocytes by H3K18 lactylation

**Fig. 7.**
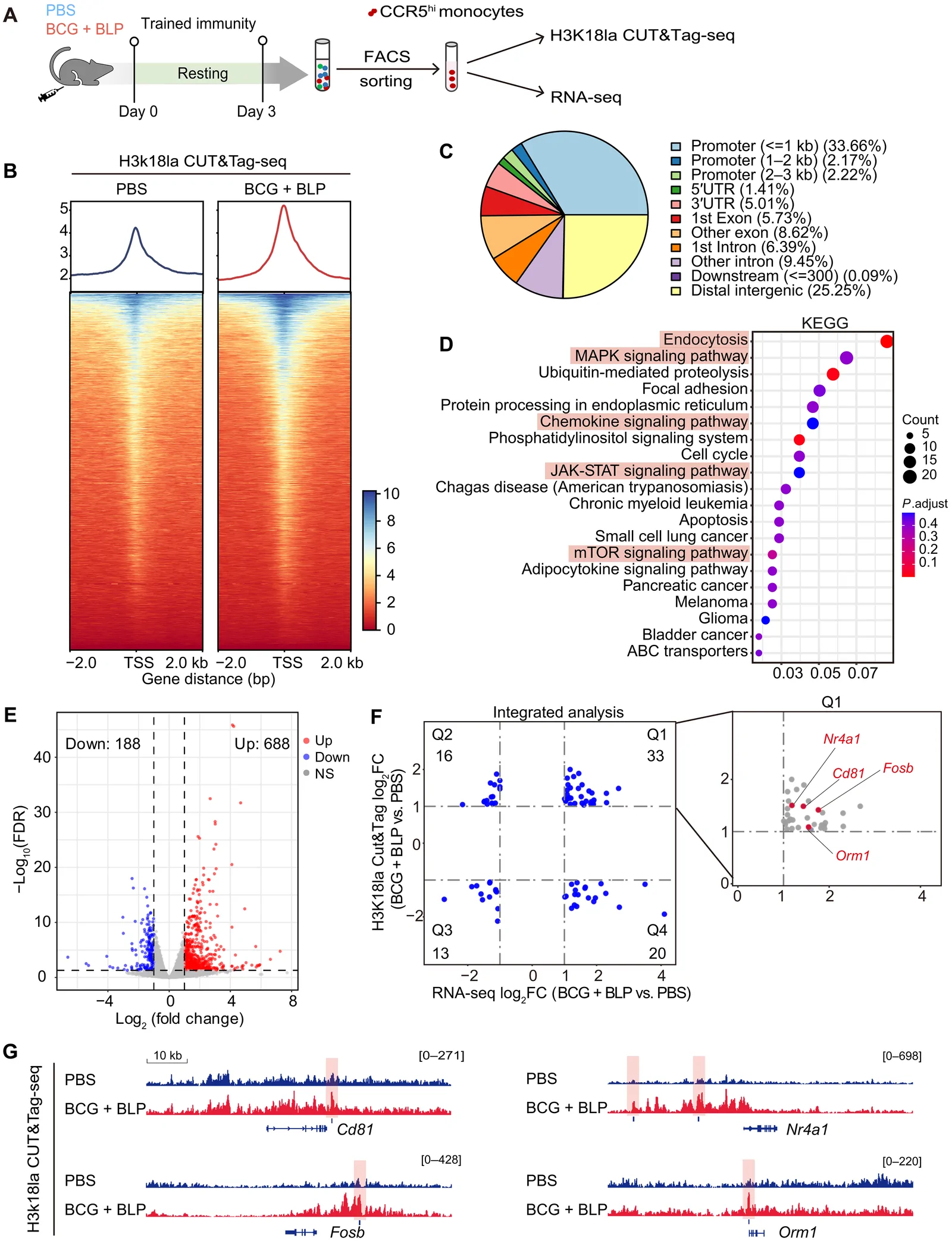
Epigenetic activation of CCR5^hi^ monocytes by H3K18 lactylation. (**A**) Schematic diagram of functional assessment. Seven-day-old mice were treated with PBS or BCG + BLP. After a 3-day resting period, bone marrow–derived CCR5^hi^ monocytes were sorted by flow cytometry and subjected to CUT&Tag analysis to map H3K18la binding sites or to RNA-seq to identify lactylation-associated downstream genes. (**B**) Heatmap showing H3K18la occupancy at TSS regions of protein-coding genes. bp, base pairs. (**C**) Genomic distribution of H3K18la peaks. UTR, untranslated region. (**D**) KEGG pathway enrichment analysis of genes with H3K18la peaks in promoter regions. MAPK, mitogen-activated protein kinase; JAK-STAT, Janus kinase–signal transducers and activators of transcription; ABC, ATP-binding cassette. (**E**) Volcano plot showing DEGs identified by RNA-seq. (**F**) Integrated analysis of CUT&Tag and RNA-seq identifying candidate H3K18la-associated downstream targets. FC, fold change. (**G**) Integrative Genomics Viewer tracks showing H3K18la enrichment at the *Cd81*, *Fosb*, *Nr4a1*, and *Orm1* loci.

To elucidate how H3K18 lactylation contributes to the functional reprogramming of CCR5^hi^ memory-like monocytes during BCG + BLP–induced trained immunity, we performed Cleavage Under Targets and Tagmentation (CUT&Tag) using an anti-H3K18la antibody, in parallel with RNA-seq to identify histone lactylation–regulated target genes (Fig. 7A). Compared to PBS controls, CCR5^hi^ monocytes from BCG + BLP–trained mice exhibited increased H3K18la enrichment at transcription start sites (TSS), with ∼40% of peaks located within promoter regions (Fig. 7, B and C). KEGG pathway analysis of promoter-associated peaks revealed enrichment in phagocytosis and proinflammatory signaling pathways, including endocytosis, mitogen-activated protein kinase, chemokine, Janus kinase–signal transducers and activators of transcription, and mTOR pathways (Fig. 7D).

RNA-seq identified 688 up-regulated genes and 188 down-regulated genes in BCG + BLP–trained CCR5^hi^ monocytes compared to PBS-treated controls (Fig. 7E). KEGG analysis of the up-regulated genes also indicated strong enrichment in pathways related to phagocytosis and inflammatory responses, such as cytokine-cytokine receptor interaction, IL-17 signaling, and the complement and coagulation cascades (fig. S7A).

By integrating CUT&Tag and RNA-seq datasets, we identified 33 DEGs that displayed notable increases in both H3K18la enrichment and transcriptional expression in BCG + BLP–trained CCR5^hi^ monocytes (Fig. 7F). Among these, *Cd81*, *Nr4a1*, *Fosb*, and *Orm1* were particularly notable. CD81 regulates monocyte adhesion, motility, activation, and signal transduction while preventing the fusion of mononuclear phagocytes (*47*–*49*). NR4A1, a key stress-response factor, modulates immune regulation and energy metabolism (*50*, *51*). FOSB, an activator protein-1 (AP-1) subunit, promotes inflammatory gene transcription (*52*, *53*). ORM1, an acute-phase protein, influences immune activity, leukocyte trafficking, and cytokine responses (*54*). These functions are consistent with the enhanced phagocytic and proinflammatory properties of CCR5^hi^ monocytes. Visualization using Integrative Genomics Viewer confirmed elevated H3K18la peaks at *Cd81*, *Nr4a1*, *Fosb*, and *Orm1* loci in BCG + BLP–trained CCR5^hi^ monocytes (Fig. 7G), correlating with their increased mRNA levels (fig. S7B). Together, these findings indicate that H3K18 lactylation promotes transcriptional activation of genes involved in phagocytosis and proinflammatory signaling in CCR5^hi^ monocytes, thereby reinforcing their effector function during trained immunity against sepsis.

### BCG + BLP induces trained CCR5^hi^ monocytes in human cord blood

**Fig. 8.**
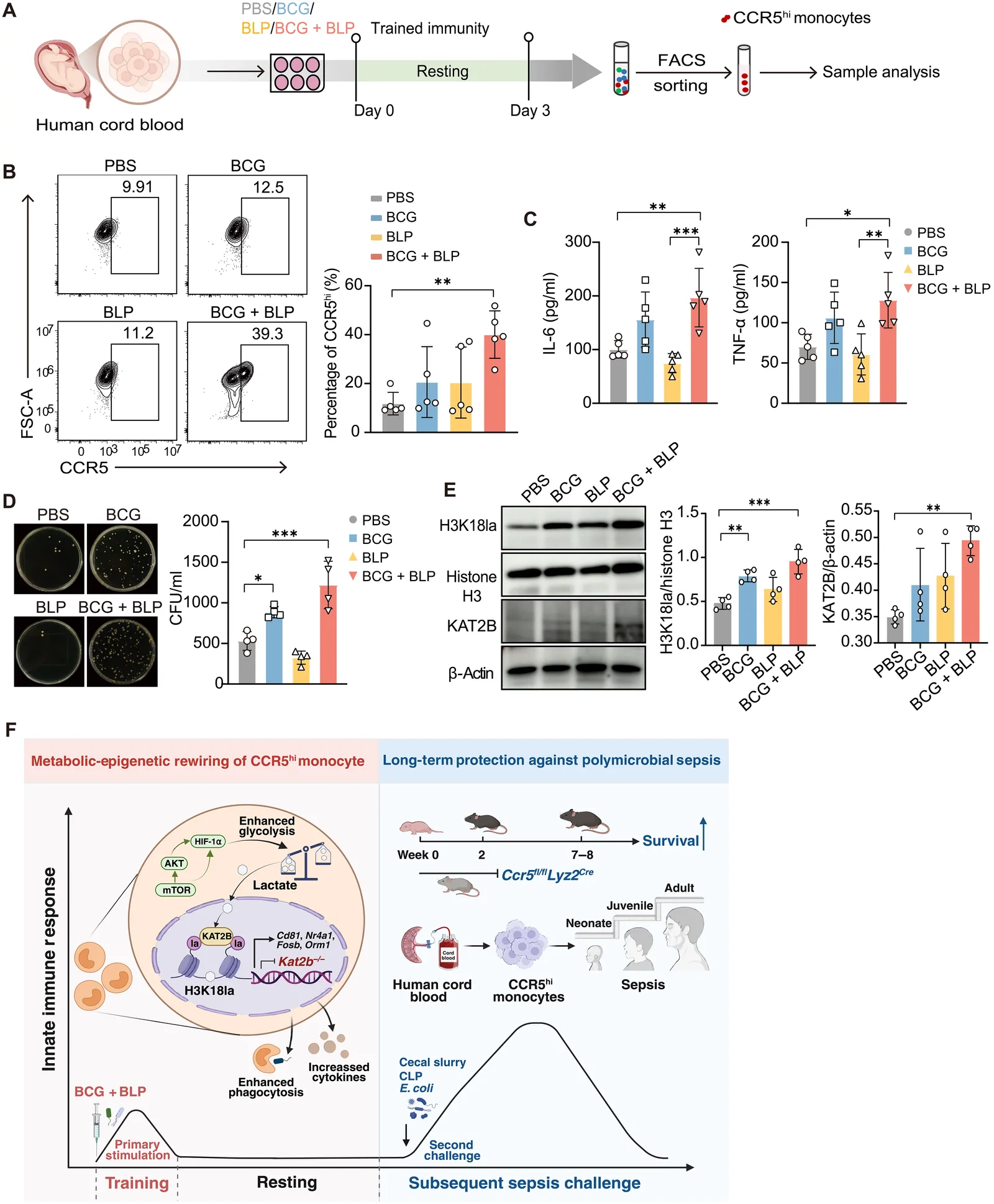
BCG + BLP induces CCR5^hi^ monocytes in human cord blood. (**A**) Schematic illustration for functional validation of CCR5^hi^ monocytes in human cord blood. Human umbilical CBMCs were treated in vitro with PBS, BCG, BLP, or BCG + BLP. After a 3-day resting period, CCR5^hi^ monocytes were sorted for analysis. (**B**) Flow cytometry analysis of CCR5^hi^ monocytes in CBMCs from the indicated groups, with quantification (*n* = 5). (**C**) IL-6 and TNF-α levels in supernatants from CCR5^hi^ monocyte 12 hours after LPS stimulation (*n* = 5). (**D**) Phagocytosis of CCR5^hi^ monocytes assessed by CFU assay after incubation with *E. coli* for 30 min (*n* = 4). (**E**) Western blot analysis of H3K18la and KAT2B expression in CCR5^hi^ monocytes from CBMCs in the indicated groups, with representative immunoblots and densitometry. H3 or actin served as loading controls (*n* = 4). (**F**) Schematic illustration of metabolic-epigenetic rewiring of CCR5^hi^ monocytes sustaining long-term trained immunity against lethal sepsis. Created in BioRender. yang, Y. (2026) https://BioRender.com/v5rt6g4. Data are representative of at least three independent experiments [(B) to (E)] and are shown as means ± SD. Statistics: one-way ANOVA [(B) to (E)]. *P < 0.05, **P < 0.01, and ***P < 0.001.

To evaluate the translational relevance of our findings, we investigated whether BCG + BLP induces similar CCR5^hi^ memory-like monocytes in humans. Human umbilical cord blood mononuclear cells (CBMCs) were isolated and treated in vitro with PBS, BCG alone, BLP alone, or BCG + BLP. After a 3-day resting period, cells were harvested for analysis (Fig. 8A).

BCG + BLP treatment increased the frequency of CCR5^hi^ monocytes within CBMCs compared to all other groups (Fig. 8B). Sorted CCR5^hi^ monocytes were then stimulated with LPS, and cytokine analysis revealed that BCG + BLP–trained CCR5^hi^ monocytes produced the highest levels of proinflammatory cytokines IL-6 and TNF-α (Fig. 8C). Functional assessment using CFU assays demonstrated enhanced bacterial phagocytosis in CCR5^hi^ monocytes from the BCG + BLP group (Fig. 8D). Consistent with the murine data, Western blot analysis showed elevated expression of KAT2B and H3K18la in BCG + BLP–trained human CCR5^hi^ monocytes (Fig. 8E). These findings support the presence of the lactate-KAT2B-H3K18la axis and CCR5^hi^ monocyte–driven trained immunity in humans, underscoring their potential role in boosting host defense early in life (Fig. 8F).

## DISCUSSION

Sepsis remains a leading cause of mortality in intensive care units, particularly among neonates, the elderly, and immunocompromised individuals (*2*–*4*). Despite extensive insights into its immunopathogenesis, therapeutic strategies targeting host immune dysfunction remain limited (*2*). In this study, we identify a coimmunization strategy using BCG and BLPs that induces robust and durable trained immunity, providing long-term protection against polymicrobial sepsis from early life into adulthood.

The World Health Organization–approved neonatal vaccine BCG is a well-recognized inducer of trained immunity with heterologous protective effects (*14*, *20*, *22*, *28*). However, its protective benefits in sepsis are often transient (*20*). To overcome this transient efficacy, we used BLP-potent immunomodulators derived from microbial membranes (*30*, *31*)—as synergistic adjuvants for BCG. BCG + BLP coimmunization significantly enhanced both the magnitude and durability of trained immunity, augmenting innate immune function in neonatal mice and conferring sustained protection across multiple sepsis models into adulthood. This strategy underscores the translational potential of early-life BCG + BLP immunoprophylaxis.

Through single-cell transcriptomics and functional validation, we identified a distinct subset of CCR5^hi^ memory-like monocytes as key effectors of this durable trained immune state. These CCR5^hi^ monocytes displayed heightened inflammatory responses, enhanced phagocytosis, and conferred protection against sepsis both endogenously and upon adoptive transfer. Genetic deletion of *Ccr5* in myeloid cells abrogated these protective effects, underscoring their indispensable role.

We observed that CCR5^hi^ monocytes with trained immunity features were recapitulated in human cord blood following ex vivo BCG + BLP stimulation. These human cells exhibited enhanced cytokine recall responses, increased phagocytic activity, and elevated KAT2B and H3K18la expression, supporting the presence of this lactate-driven trained immunity program across species. Although these human data are based on ex vivo stimulation of a limited number of cord blood samples, they provide proof-of-concept evidence that the lactate-KAT2B-H3K18la axis may operate in human neonatal monocytes. Further validation in larger, well-characterized human cohorts and clinically relevant settings will be required to establish the generalizability and physiological significance of this pathway.

Mechanistically, we uncovered a metabolic-epigenetic circuit in CCR5^hi^ monocytes that links glycolytic rewiring to durable functional reprogramming. Coimmunization with BCG + BLP reprogrammed these cells toward enhanced glycolysis and lactate accumulation. Beyond its conventional role as a metabolic byproduct, lactate served as a signaling metabolite that drove KAT2B-dependent histone H3K18 lactylation, thereby establishing long-term transcriptional priming. This epigenetic mark enhanced the expression of immune effector genes, including *Cd81*, *Nr4a1*, *Fosb*, and *Orm1*, thereby reinforcing the antimicrobial and inflammatory phenotype of trained CCR5^hi^ monocytes. Targeted deletion of *Kat2b* abolished histone lactylation and abrogated the protective effects of trained immunity, establishing KAT2B as a critical epigenetic writer in this pathway. Notably, the persistence of protection for several weeks after immunization suggests that BCG + BLP–induced trained immunity is durable and may be associated with progenitor-level reprogramming (fig. S8). Collectively, these findings identify a metabolic-epigenetic program sustaining trained immunity in CCR5^hi^ monocytes.

Our findings align with recent work demonstrating that BCG-induced lactate mediates histone lactylation and modulates long-term inflammation (*45*). Although previous studies have implicated other enzymes such as P300 and KAT2A in histone lactylation (*45*, *46*, *55*), our data highlight KAT2B as the dominant lactyltransferase responsible for durable immune reprogramming in the context of trained immunity. This deepens our mechanistic understanding of the immunometabolic control of innate memory.

Several aspects warrant further investigation. First, although CCR5^hi^ monocytes emerged as the dominant effector population, BCG + BLP may also influence other innate immune compartments, including macrophages, neutrophils, NK cells, or hematopoietic progenitors. Second, our in vivo validation was based primarily on bacterial abdominal sepsis models, including CS, CLP, and *E. coli* challenge; whether this trained immunity program extends to viral, fungal, or tissue-specific infectious contexts remains to be determined. Third, although our data support a central role for the lactate-KAT2B-H3K18la axis, additional metabolic inputs and lactylation-associated regulators, including potential readers and erasers, may further shape this epigenetic program (fig. S9). Last, the translational relevance and long-term safety of sustained CCR5^hi^ monocyte expansion require further validation in larger human cohorts, adult peripheral blood samples, and clinically relevant disease settings.

In summary, our findings identify a lactate-KAT2B-H3K18la axis in CCR5^hi^ monocytes that contributes to long-term trained immunity and protection against sepsis. This work further advances our understanding of the metabolic-epigenetic regulation of trained immunity and suggests a potentially translatable strategy to enhance early-life immune protection against sepsis and possibly other infectious diseases.

## MATERIALS AND METHODS

### Reagents

The sources of all reagents and materials used in this study are listed in table S1.

### Umbilical cord blood collection

This study was approved by the Ethics Review Committee of Children’s Hospital of Soochow University (approval no. 2025CS124) and conducted in accordance with the Declaration of Helsinki. Umbilical cord blood samples were obtained from nine healthy neonates at Children’s Hospital of Soochow University (the Affiliated Zhangjiagang Hospital). Written informed consent was obtained from the parents or legal guardians of all participating neonates before sample collection. Exclusion criteria included maternal diabetes mellitus, intrauterine infections, hypertensive disorders of pregnancy (e.g., preeclampsia), and any maternal or neonatal condition potentially affecting immune development (table S2).

### Mice

All animal experiments were approved by the Ethics Committee of Soochow University (approval no. SDAU 20231228A01) and conducted in accordance with the Guide for the Care and Use of Laboratory Animals (National Institutes of Health Publication No. 85-23, revised 1996). C57BL/6 mice were purchased from JOINN Laboratories (Suzhou, China; license no. SCXK 2022-0005) and housed under specific pathogen–free conditions. *Kat2b* knockout mice were obtained from Cyagen Biosciences (Suzhou, China). *Lyz2*^+/cre^ and *Ccr5*^fl/fl^ mice were provided by the Institutes for Translational Medicine, Suzhou Medical College, Soochow University (Suzhou, Jiangsu, China).

### In vivo induction of trained immunity and sepsis models

To evaluate the long-term effects of trained immunity in polymicrobial sepsis, 7-day-old neonatal mice were intraperitoneally injected with PBS (control), BCG (250 μg/g), BLP (5 μg/g), or BCG + BLP. Mice were rested for 3, 14, or 49 days postimmunization.

For polymicrobial sepsis induction, both CS and CLP models were used. In neonatal mice, the cecum is not readily distinguishable from the small intestine, rendering ligation and puncture technically infeasible. In addition, surgical intervention and anesthesia are associated with high perioperative mortality and maternal cannibalization, thereby compromising experimental reproducibility. The CS model, which enables the induction of systemic polymicrobial sepsis without surgical manipulation, is therefore widely used in neonatal settings. In contrast, in adult mice, the CLP model is technically feasible and represents the most extensively validated model of intra-abdominal polymicrobial sepsis, closely recapitulating the progression of clinical disease. Accordingly, the CS model was used in neonatal and juvenile mice, whereas the CLP model was applied in adult mice (*32*, *56*–*59*).

For CS-induced sepsis, cecal contents from adult donor mice were suspended in 5% dextrose to 100 mg/ml and used within 2 hours. Neonatal and juvenile mice were intraperitoneally injected with a cecal content suspension at a dose of 1.0 mg/g body weight.

For the CLP model, adult mice were anesthetized with pentobarbital, and the cecum was ligated below the ileocecal valve and punctured once with a 20G needle. Sham-operated mice underwent laparotomy without ligation or puncture. Resuscitation was performed via subcutaneous injection of 1 ml of prewarmed saline per 20 g body weight. For the single-bacterial sepsis model, 6- to 8-week-old mice were intraperitoneally injected with *E. coli* (American Type Culture Collection 25922; 4 × 10^6^ CFU).

Peripheral blood was collected at 1 hour postsepsis induction under anesthesia for cytokine analysis. At 24 hours postsepsis, mice were euthanized, and blood and organs including heart, liver, spleen, and lungs were harvested. Lung, liver, and spleen were homogenized. Homogenates were serially diluted (10-fold in PBS), and 100-μl aliquots were plated onto LB agar plates. After 24 hours of aerobic incubation at 37°C with 5% CO_2_, intra-organ bacterial loads were computed from colony counts and then photographed. Major organs were fixed and stained with hematoxylin and eosin (H&E). Mice were monitored for 96 hours for survival analysis.

### In vitro induction of trained immunity

Umbilical CBMCs were isolated, seeded, and stimulated for 24 hours with BCG (5 μg/ml), BLP (100 ng/ml), or BCG + BLP. After PBS washes, cells were cultured in fresh medium for 3 days (resting phase) and then restimulated with LPS (100 ng/ml) for 12 hours. Supernatants were collected for cytokine analysis. For phagocytosis assays, cells were incubated with *E. coli* for 30 min.

### Cytokine and metabolite quantification

The concentrations of TNF-α and IL-6 in mouse serum or cell supernatants were determined using commercially available enzyme-linked immunosorbent assay kits (DAKEWE), following the manufacturer’s instructions. Levels of LDH and ALT were measured using an automated biochemical analyzer (Hitachi High-Tech). Intracellular lactate concentrations were quantified with the Lactic Acid Content Assay Kit (Sigma-Aldrich) according to the manufacturer’s protocol.

### Isolation of PBMCs

Whole blood collected in EDTA tubes was diluted 1:1 with PBS and layered onto separation medium (Solarbio). After centrifugation at 500*g* for 30 min, the PBMC layer was collected, washed, and treated with red blood cell lysis buffer.

### Histology staining and pathological assessments

Mouse tissues were fixed in 4% paraformaldehyde, embedded in paraffin, and sectioned at 4-μm thickness. Following deparaffinization and rehydration, sections were subjected to H&E staining according to standard procedures. Histopathological evaluation was performed independently by two investigators blinded to the experimental groups. Semiquantitative scoring systems were applied to assess tissue injury in each organ, as described below.

Liver injury was graded on a 0-4 scale as previously described. Grade 0 indicates normal hepatic architecture; grade 1, mild hepatic congestion, cytoplasmic vacuolization, or isolated necrotic cells; grade 2, pathological changes involving <30% of the examined area; grade 3, injury involving up to 60% of the microscopic field; and grade 4, extensive hemorrhage and necrosis involving >60% of the section (*60*, *61*).

Lung injury was assessed on the basis of interstitial inflammation, alveolar wall thickening, inflammatory cell infiltration, alveolar collapse, congestion, and edema. Each parameter was scored on a 0-4 scale (0, no injury; 1, mild; 2, moderate; 3, severe; 4, very severe). The total lung injury score was calculated as the sum of all individual parameters (*62*).

Renal tubular injury was evaluated using a semiquantitative scoring system as follows: 0, normal renal morphology; 1, loss of brush border in <25% of tubular epithelial cells with intact basement membrane; 2, loss of brush border in >25% of tubular epithelial cells with basement membrane thickening; 3, presence of tubular cast formation and necrosis involving up to 60% of tubules; and 4, extensive tubular necrosis involving >60% of tubular structures (*63*).

Myocardial injury was evaluated on the basis of established histopathological criteria and scored as follows: 0, no detectable damage; 1 (mild), interstitial edema and focal necrosis; 2 (moderate), widespread cardiomyocyte swelling and necrosis; 3 (severe), necrosis with contraction band formation, neutrophil infiltration, and capillary compression; and 4 (very severe), diffuse necrosis with contraction bands, dense inflammatory infiltration, capillary compression, and hemorrhage (*64*).

### scRNA-seq and analysis

PBMCs from six to eight mice per group were pooled before single-cell library preparation to ensure sufficient cell numbers and to minimize interindividual variability. All samples were processed in parallel under identical experimental conditions. Because of experimental constraints, independent biological replicates were not included at the single-cell level, which is acknowledged as a limitation.

Single-cell suspensions were adjusted to approximately 1000 cells/μl. Cells were subsequently loaded into Gel Beads within the Chromium instrument (10x Genomics), where cell lysis and barcoded reverse transcription of RNA were performed. The resulting cDNA was amplified and used to construct sequencing libraries, which were sequenced on the Illumina NovaSeq 6000 platform by Gene Denovo Biotechnology Co. (Guangzhou, China). Raw sequencing reads were aligned to the mouse reference genome (GRCm38/mm10) using Cell Ranger (10x Genomics).

Downstream analysis was carried out using the Seurat package in R. Quality control and filtering were performed to remove low-quality and abnormal cells, including doublets, based on the following criteria: gene count between 200 and 6000, UMI count <33,000, and mitochondrial gene content <10%.

Data normalization and integration across samples were conducted using the Harmony algorithm to correct for batch effects. Principal components analysis was applied for dimensionality reduction, followed by unsupervised clustering using Seurat. Cell clusters were visualized using t-distributed stochastic neighbor embedding (t-SNE).

Cell types were annotated using SingleR for automated classification, which was further refined through manual validation based on canonical marker gene expression. Differential gene expression analysis between clusters was performed using the Wilcoxon rank sum test. Genes were considered significantly up-regulated if they met the following criteria: expressed in more than 25% of cells in either group, *P* value ≤ 0.01, and log_2_ fold change ≥ 0.58. Functional enrichment analysis of DEGs was conducted using the clusterProfiler package in R.

### Flow cytometry and cell sorting

PBMCs were adjusted to a final concentration of 1 × 10^7^ cells/ml and stained with antibodies at 100:1 (cell:antibody), analyzed using BD Biosciences cytometers. For mouse samples, the antibody panel included phycoerythrin (PE) anti-mouse/human CD11b, BV421 rat anti-mouse Ly-6G, allophycocyanin (APC) rat anti-mouse Ly-6C, and PE/Cyanine7 anti-mouse CD195 (CCR5). For human samples, antibodies included PE anti-human CD45, APC anti-human CD14, fluorescein isothiocyanate (FITC) anti-human CD16, and Brilliant Violet 421 anti-human CD195. Staining was done on ice for 30 min in the dark. The flow cytometric data were analyzed with FlowJo software (version 10.8.1).

Bone marrow and cord blood cells were stained similarly and sorted for CCR5^hi^ monocytes. For bone marrow staining, the antibody panel included peridinin-chlorophyll-protein complex (PerCP)/Cyanine5.5 anti-mouse CD45, PE anti-mouse/human CD11b, BV421 rat anti-mouse Ly-6G, APC rat anti-mouse Ly-6C, and PE/Cyanine7 anti-mouse CD195 (CCR5). For umbilical cord blood PBMCs, antibodies included PE anti-human CD45, APC anti-human CD14, FITC anti-human CD16, and Brilliant Violet 421 anti-human CD195 (CCR5). Sorting was performed on BD FACSAria.

### Phagocytosis assay

CCR5^hi^ and CCR5^lo^ monocytes were incubated with *E. coli* at a multiplicity of infection of 60 for 30 min at 37°C. After infection, cells were washed three times with PBS containing gentamicin (25 μg/ml; MedChemExpress [MCE], USA) to eliminate extracellular bacteria. The monocytes were then lysed with 0.3% Triton X-100 (Sigma-Aldrich) for 5 min at room temperature. The resulting lysates were then serially diluted 10-fold in PBS, and 100-μl aliquots of appropriate dilutions were aseptically plated onto LB agar plates. After overnight incubation at 37°C under aerobic conditions, bacterial CFUs were quantified manually by two laboratory technicians. The LB agar plates were then photographed for permanent experimental documentation.

For phagosome acidification, CCR5^lo^ and CCR5^hi^ monocytes were incubated with the pHrodo BioParticles Phagocytosis Kit (Invitrogen, USA) for 20 min according to the manufacturer’s instructions. Phagosome acidification, indicative of phagocytic activity, was analyzed by flow cytometry.

### Adoptive transfer

CD45^+^CD11b^+^Ly6G^−^Ly6C^+^CCR5^hi^ monocytes were sorted from the bone marrow of BCG + BLP or PBS-treated mice via flow cytometry. Recipient mice were intravenously injected via the tail vein with 100 μl of PBS (NC), 1 × 10^6^ sorted monocytes from PBS-treated donors, or 1 × 10^6^ sorted monocytes from BCG + BLP–treated donors. Two hours posttransfer, polymicrobial sepsis was induced. Mouse survival was subsequently monitored, and biological samples were collected for downstream analyses.

### Seahorse experiments

Sorted CCR5^hi^ monocytes were seeded in XF24 cell culture plates (2 × 10^5^ cells per well). Cells were then stimulated with LPS (100 ng/ml; Sigma-Aldrich) for 12 hours. The Seahorse XFe24 Flux Assay Kit (Agilent Technologies) was used. For the glycolysis stress test, glucose (10 mM), oligomycin (1 μM), and 2-DG (50 mM) were sequentially loaded into the cartridge ports. For the mitochondrial stress test, oligomycin (1.5 μM), carbonyl cyanide *p*-trifluoromethoxyphenylhydrazone (1.5 μM), and rotenone/antimycin A (0.5 μM) were added. ECAR and OCR were measured using a Seahorse XFe24 Analyzer.

### Western blot analysis

Total protein was extracted by lysing samples in radioimmunoprecipitation assay buffer (Beyotime Biotechnology), followed by centrifugation to collect the supernatant. Protein samples were separated by SDS–polyacrylamide gel electrophoresis and transferred onto polyvinylidene difluoride membranes (Millipore). Membranes were blocked with NcmBlot Blocking Buffer (NCM Biotech) at room temperature for 10 min and incubated overnight at 4°C with the appropriate primary antibodies. After washing, membranes were incubated with secondary antibodies for 1 hour at room temperature. Protein signals were visualized using enhanced chemiluminescence reagent (Millipore). Full, uncropped Western blots with loading controls and molecular weight markers are provided in data S1.

### CUT&Tag assay

The CUT&Tag assay was performed using the Hyperactive Universal CUT&Tag Assay Kit for Illumina Pro (TD904, Vazyme) following the manufacturer’s instructions. DNA was extracted and amplified with i5 and i7 primers from the TruePrep Index Kit V2 for Illumina (TD202, Vazyme). Libraries were purified using VAHTS DNA Clean Beads (N411, Vazyme) and sequenced at Nanjing Jiangbei New Area (Nanjing, China).

After sequencing, high-quality reads were filtered and aligned to the mouse reference genome (GRCm38-mm10). Peaks were identified on the basis of the alignment results. Subsequent analyses included functional annotation of genes associated with the peaks, differential peak analysis between groups, and enrichment analysis of genes linked to differential peaks.

### Bulk RNA-seq

Total RNA was extracted using the TRIzol and assessed using Agilent 2100 Bioanalyzer. mRNA was enriched using the VAHTS mRNA Capture Beads Kit, followed by library construction with the VAHTS Universal V8 RNA-seq Library Prep Kit for Illumina. The quality of the libraries was evaluated using the Qubit 4.0 fluorometer and the ABI QuantStudio 12K fluorescence quantitative polymerase chain reaction system. Sequencing was performed on the Illumina NovaSeq 6000 platform by Nanjing Jiangbei New Area (Nanjing, China). Raw data were subjected to standard RNA-seq analysis, including quality control, differential gene expression analysis, and enrichment analysis.

### Statistical analysis

Unless otherwise specified, the quantitative data are presented as mean ± SD. All data met the assumptions of the tests, such as normal distribution. Data normality was assessed using the Shapiro-Wilk test. The means of two groups were compared using unpaired Student’s *t* tests. Analysis of variance (ANOVA) was used to test for differences among three or more groups, and if the ANOVA showed differences, we used Tukey’s post hoc test to identify pairs with differences. Differences in mortality rates between groups were evaluated using a log-rank test. A two-tailed *P* value of <0.05 was considered statistically significant. The exact value of *n* for each figure is provided in the respective figure legends.
