# Supplementary material for: Metabolic-epigenetic rewiring of CCR5hi monocytes sustains long-term trained immunity against lethal sepsis
Source: Sci Adv. 2026 Aug 21;12(34):eaee1634. doi: 10.1126/sciadv.aee1634 (PMC13496178; doi:10.1126/sciadv.aee1634)

Science Advances
AAAS

Supplementary Materials for

Metabolic-epigenetic rewiring of CCR5^{hi} monocytes sustains long-term trained immunity against lethal sepsis

Lingqi Xu *et al.*

Corresponding author: Jian Wang, wj196312@vip.163.com; Daolin Tang, daolin.tang@utsouthwestern.edu; Huiting Zhou, htzhou@suda.edu.cn

Sci. Adv. **12**, eaee1634 (2026)
DOI: 10.1126/sciadv.aee1634

This PDF file includes:

Figs. S1 to S9
Tables S1 and S2
Data S1

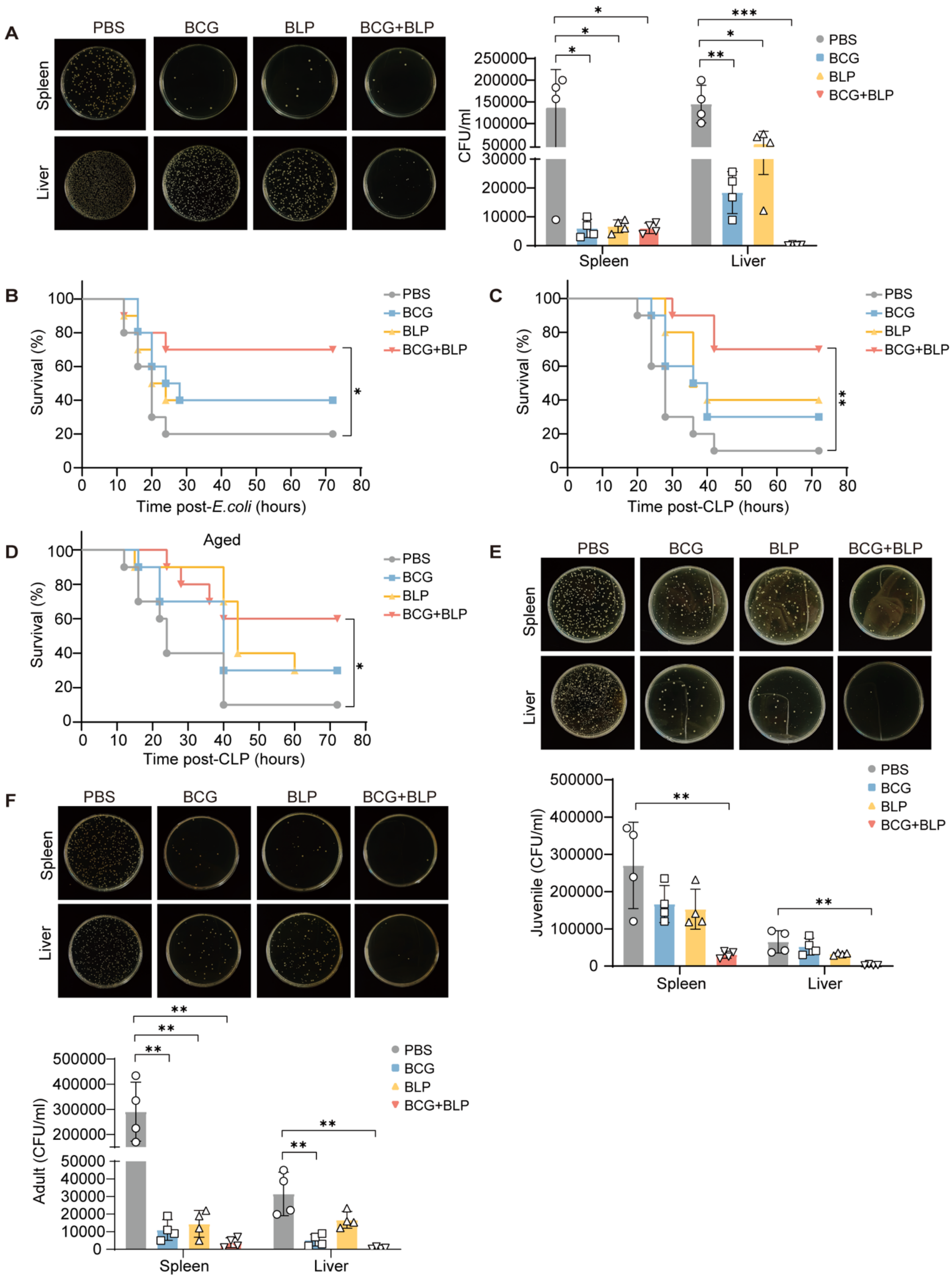
A
PBS
BCG
BLP
BCG+BLP
Spleen
Liver
CFU/ml
250000
200000
150000
100000
50000
30000
20000
10000
0
*
**
***
B
Survival (%)
Time post-E.coli (hours)
C
Time post-CLP (hours)
D
Aged
Time post-CLP (hours)
E
Juvenile (CFU/ml)
500000
400000
300000
200000
100000
F
Adult (CFU/ml)
40000

Fig. S1. BCG+BLP-induced trained immunity confers long-term antimicrobial protection, related to Fig. 1. (**A**) Bacterial loads in spleen and liver 24 hours after sepsis induction at 3 days post-immunization. 7-day-old mice were treated with PBS, BCG, BLP, or BCG+BLP, followed by polymicrobial sepsis induction with cecal slurry (CS). Representative plates and quantification are shown (n = 4 mice per group). (**B** and **C**) Survival of 6- to 8-week-old mice treated with PBS, BCG, BLP, or BCG+BLP after sepsis induced by *E. coli* (4×10^6 CFU) (**B**) or CLP (**C**) at 3 days post-immunization (n = 10 mice per group). (**D**) Survival of aged mice (18 months old) after CLP-induced sepsis at 3 days post-immunization (n = 10 mice per group). (**E** and **F**) Bacterial loads in spleen and liver 24 hours after sepsis induction at 2 weeks (**E**) or 7 weeks (**F**) post-immunization, with representative plates and quantification (n = 4 mice per group). Data are representative of at least three independent experiments (**A**, **E** and **F**) and are shown as means ± SD. Statistics: one-way ANOVA (**A**, **E** and **F**) and log-rank test (**B** to **D**). *$P < 0.05$, **$P < 0.01$, ***$P < 0.001$, ****$P < 0.0001$.

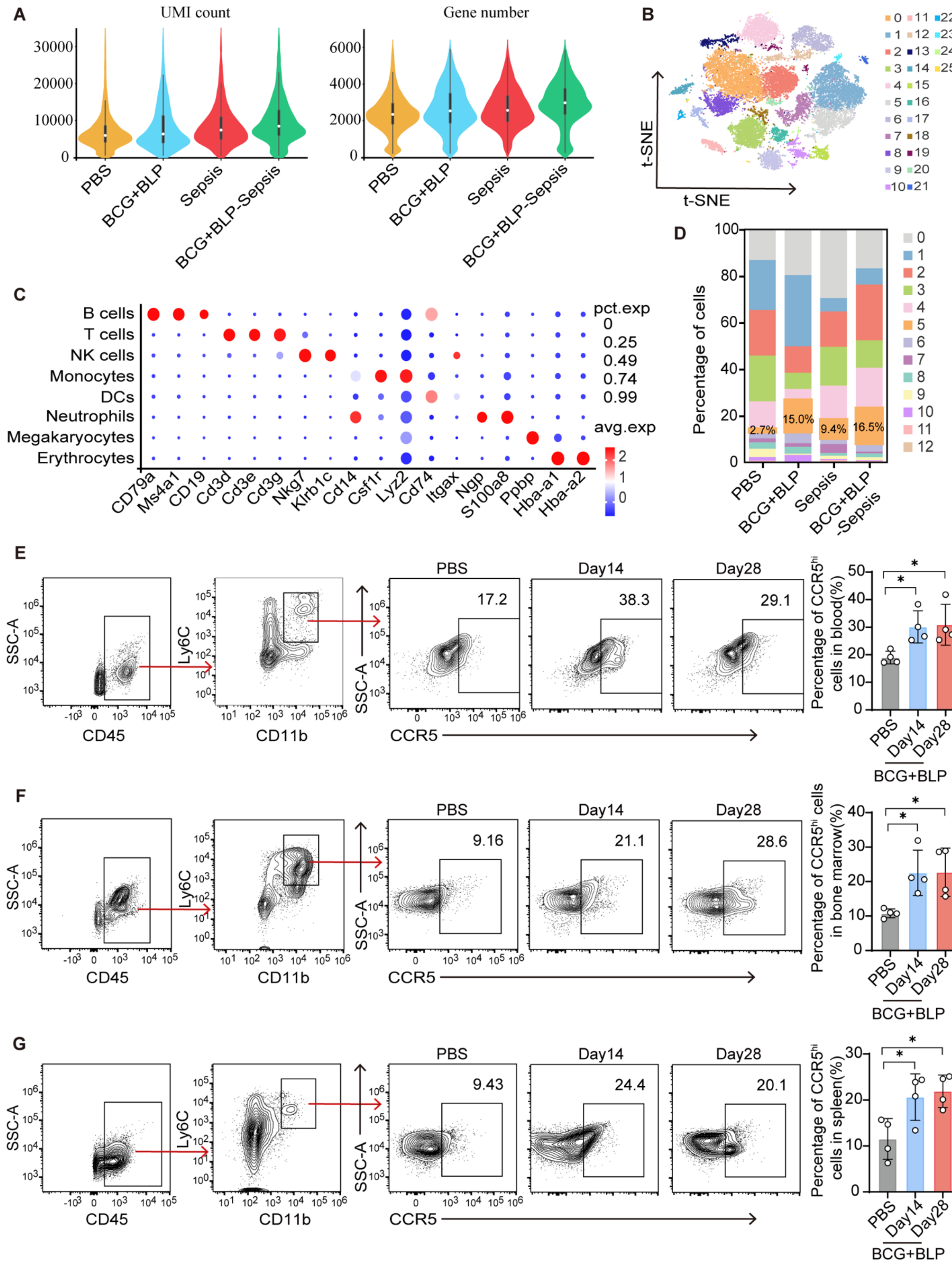

A
UMI count
Gene number
PBS
BCG+BLP
Sepsis
BCG+BLP-Sepsis
B
t-SNE
C
B cells
T cells
NK cells
Monocytes
DCs
Neutrophils
Megakaryocytes
Erythrocytes
pct.exp
avg.exp
CD79a
Ms4a1
CD19
Cd3d
Cd3e
Cd3g
Nkg7
Klrb1c
Cd14
Csf1r
Lyz2
Cd74
Itgax
Ngp
S100a8
Ppbp
Hba-a1
Hba-a2
D
Percentage of cells
2.7%
15.0%
9.4%
16.5%
E
SSC-A
CD45
Ly6C
CD11b
CCR5
PBS
Day14
Day28
17.2
38.3
29.1
Percentage of CCR5hi cells in blood(%)
BCG+BLP
F
9.16
21.1
28.6
Percentage of CCR5hi cells in bone marrow(%)
G
9.43
24.4
20.1
Percentage of CCR5hi cells in spleen(%)

Fig. S2. Single-cell atlas of immune cells during BCG+BLP – induced trained immunity, related to Fig. 2. (**A**) Violin plots showing average unique molecular identifier (UMI) counts and gene numbers in the indicated scRNA-seq groups. Each dot represents a cell. (**B**) t-SNE plots of 31,132 immune cells allocated into 26 clusters from PBMCs of wild-type mice in the indicated groups. (**C**) Heatmap showing selected cluster marker genes and cell-type annotation labeled. (**D**) Bar plots showing the distribution of 13 monocyte compartments from PBMCs of wild-type mice in the indicated groups. (**E** to **G**) Flow cytometric analysis of $CD11b^{+}Ly6C^{+}CCR5^{hi}$ monocytes in blood (**E**), bone marrow (**F**) and spleen (**G**) with quantification (n = 4 mice per group). Data are representative of at least two independent experiments (**E** to **G**) and are shown as means ± SD. Statistics: one-way ANOVA (**E** to **G**). *$P < 0.05$, **$P < 0.01$, ***$P < 0.001$, ****$P < 0.0001$.

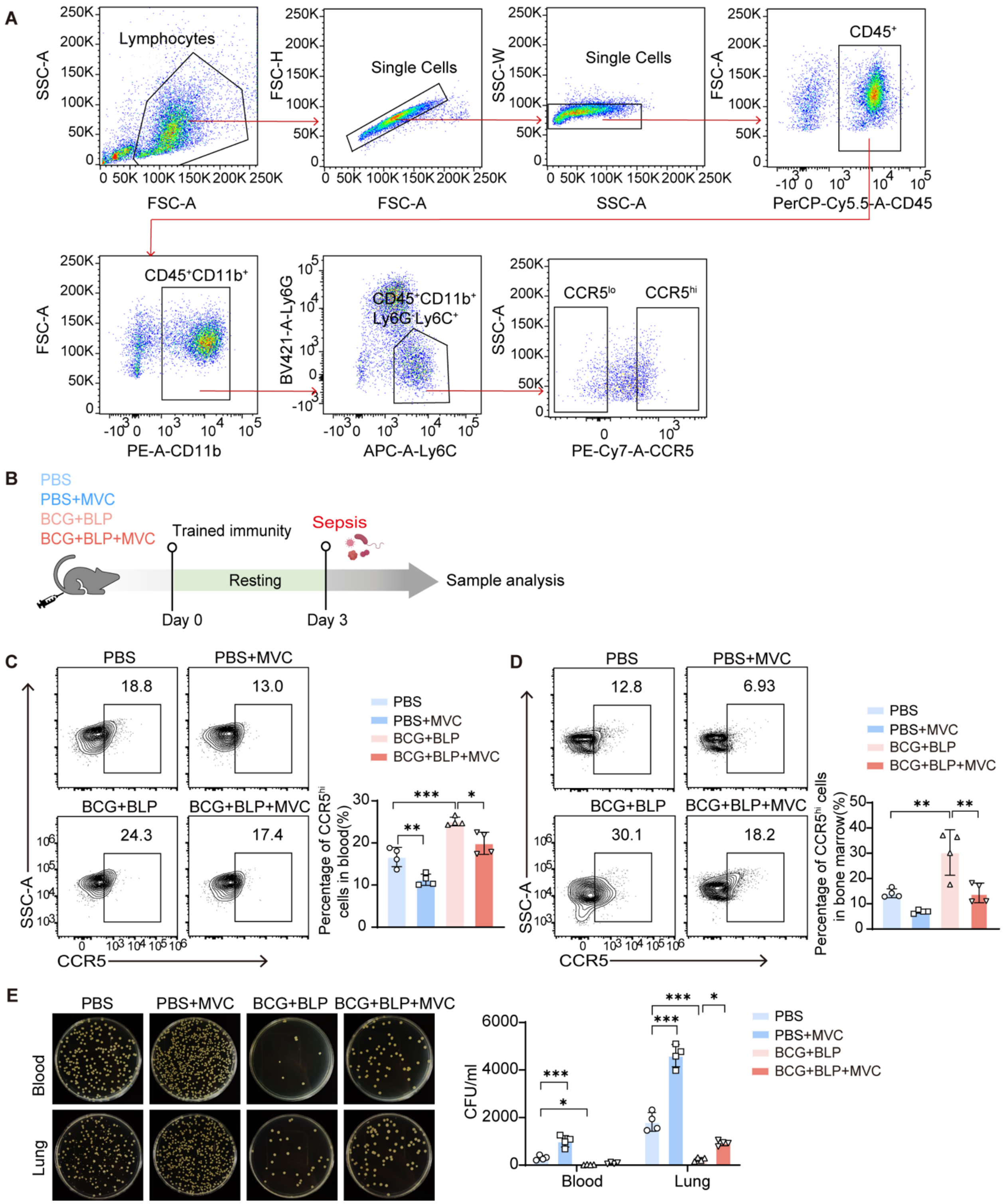

Fig. S3. Gating strategies for flow cytometry analysis of CCR5^{hi} monocytes, related to Fig. 3. (**A**) Flow cytometry gating strategy for identifying CCR5^{hi} monocytes in mice. (**B**) Schematic illustration for validating the functional role of CCR5^{hi} monocytes. 7-day-old mice were treated

with PBS or BCG+BLP, with or without the CCR5 antagonist MVC. After a 3-day resting period, frequency of $CCR5^{hi}$ monocytes were analyzed by flow cytometry in one cohort, whereas a separate cohort underwent polymicrobial sepsis induction for assessment of bacterial burdens in organs. (**C** and **D**) Flow cytometric analysis of $CD11b^{+}Ly6C^{+}CCR5^{hi}$ monocytes in blood (**C**) and bone marrow (**D**), with quantification (n = 4 mice per group). (**E**) Bacterial loads in blood and lung 24 hours after sepsis induction, with representative plates and quantification (n = 4 mice per group). Data are representative of at least three independent experiments (**C** to **E**) and are shown as means ± SD. Statistics: one-way ANOVA (**C** to **E**). $*P < 0.05$, $**P < 0.01$, $***P < 0.001$, $****P < 0.0001$.

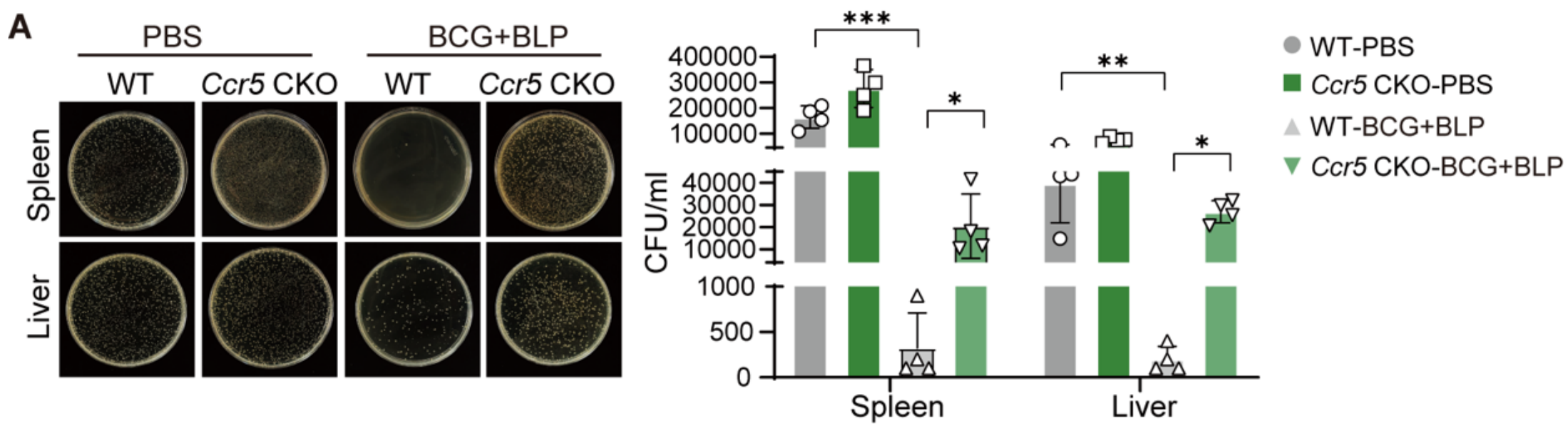

Fig. S4. CCR5 is critical for the protective effects of BCG+BLP-induced trained immunity against sepsis, related to Fig. 4. (**A**) Bacterial loads in spleen and liver 24 hours after sepsis induction. 6- to 8-week-old WT and *Ccr5* CKO mice were immunized with PBS or BCG+BLP. After a 3-day resting period, polymicrobial sepsis was induced, and bacterial burden was assessed. Representative plates and quantification are shown (n = 4 mice per group). Data are representative of at least three independent experiments and are shown as means ± SD. Statistics: one-way ANOVA. *$P < 0.05$, **$P < 0.01$, ***$P < 0.001$, ****$P < 0.0001$.

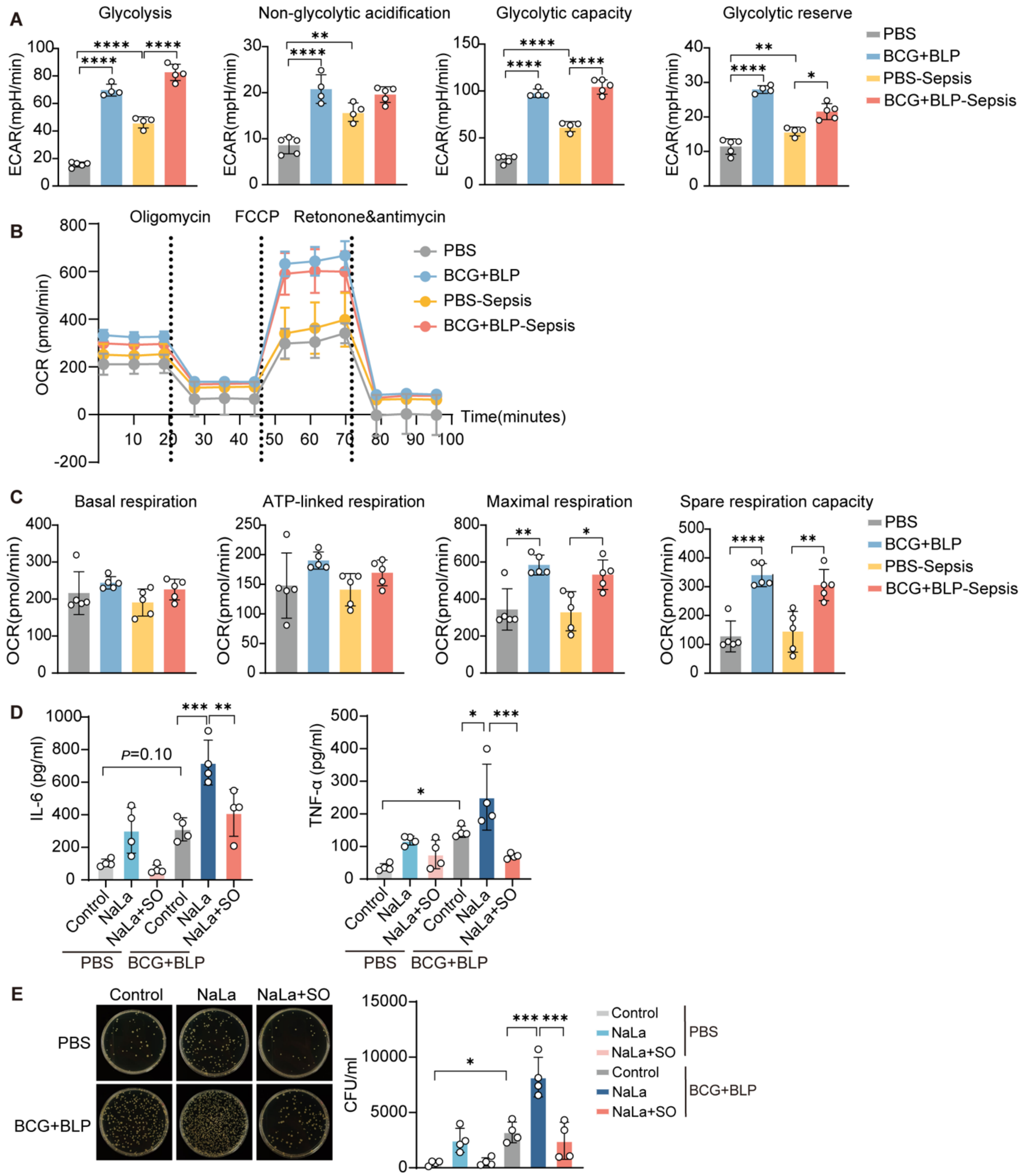

Fig. S5. BCG+BLP-induced trained immunity activates aerobic glycolysis in CCR5^{hi} monocytes, related to Fig. 5. (**A-C**) 7-day-old mice were treated with PBS or BCG+BLP. After a 3-day resting period, bone marrow-derived CCR5^{hi} monocytes were isolated and stimulated *ex vivo* with LPS for 12 hours. Metabolic changes in glycolysis and OXPHOS were assessed using

Seahorse XF analysis. (**A**) Glycolysis in CCR5^{hi} monocytes (n = 5). (**B**) Seahorse analysis of mitochondrial OXPHOS in CCR5^{hi} monocytes from the indicated groups. (**C**) Quantification of OXPHOS parameters (n = 4-5). (**D** and **E**) 7-day-old mice were treated with PBS or BCG+BLP, with or without the LDH inhibitor sodium oxamate (SO) and/or sodium lactate (NaLa). Sorted CCR5^{hi} monocytes were stimulated *ex vivo* with LPS or *E. coli.* IL-6 and TNF-α levels were measured after LPS stimulation (**D**), and phagocytosis was assessed by CFU assay after *E. coli* incubation (**E**) (n = 4). ECAR, extracellular acidification rate; OCR, oxygen consumption rate. Data are representative of at least three independent experiments and are shown as means ± SD. Statistics: one-way ANOVA. *$P < 0.05$, **$P < 0.01$, ***$P < 0.001$, ****$P < 0.0001$.

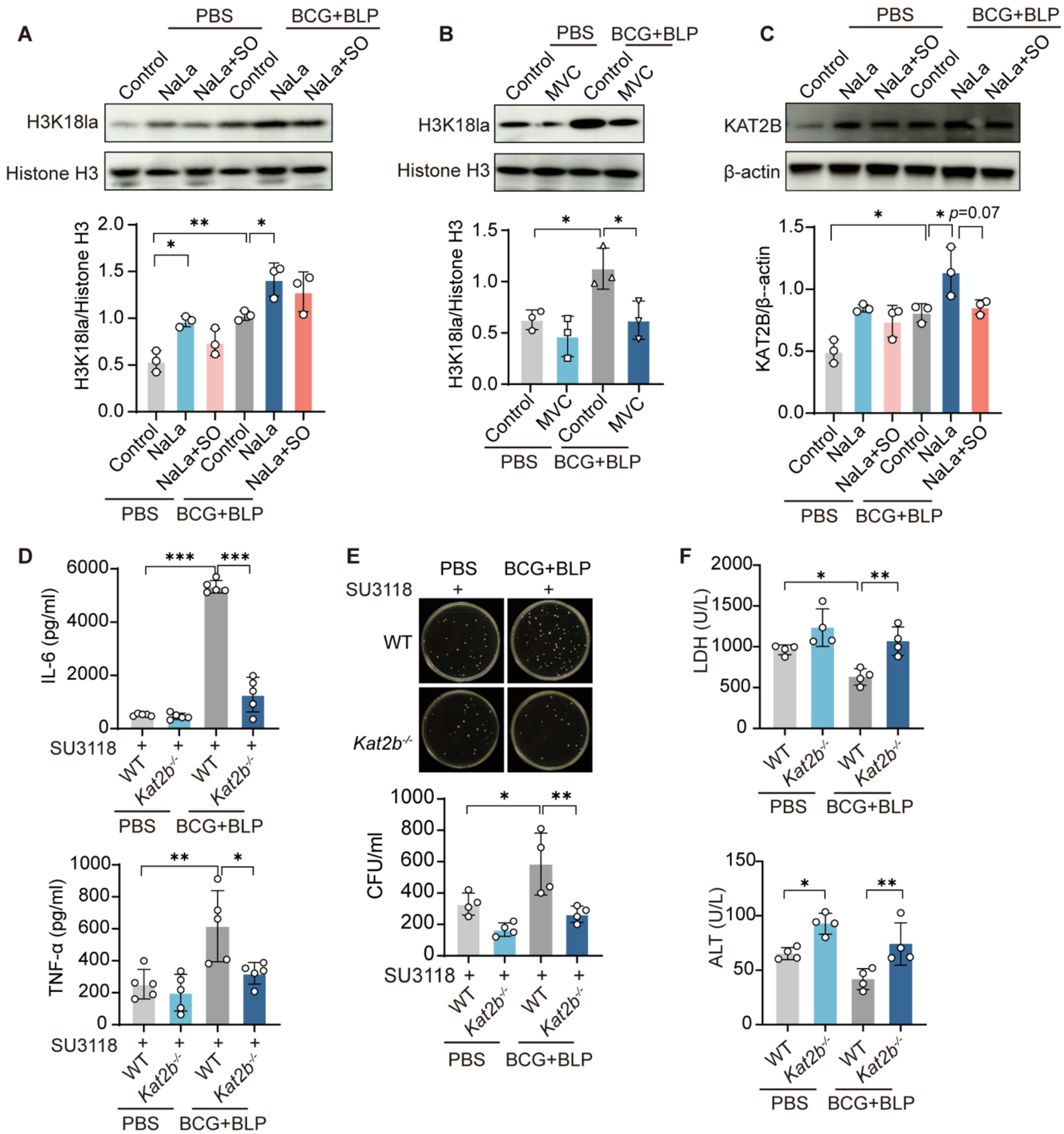

Fig. S6. Lactate-driven H3K18 lactylation requires KAT2B to program immune memory in CCR5^{hi} monocytes, related to Fig. 6. (**A** and **B**) Western blot analysis of histone lactylation in CCR5^{hi} monocytes from the indicated groups. 7-day-old mice were treated with the indicated reagents. After a 3-day resting period, CCR5^{hi} monocytes were isolated from bone marrow. Histone H3 served as loading control. Representative immunoblots and densitometry are shown (n = 3). (**C**) Western blot analysis of KAT2B expression in CCR5^{hi} monocytes, with actin as loading control. Representative immunoblots and densitometry are shown (n = 3). (**D**) IL-6 and

TNF-α levels in the supernatants of $CCR5^{hi}$ monocytes from WT or *Kat2b*$^{-/-}$ mice treated with PBS or BCG+BLP in the presence of SU3118, measured 12 hours after LPS stimulation (n = 5). (**E**) Phagocytosis of $CCR5^{hi}$ monocytes from WT or *Kat2b*$^{-/-}$ mice treated with PBS or BCG+BLP in the presence of SU3118, assessed by CFU assay after *E. coli* incubation (n = 4). (**F**) Serum LDH and ALT levels in WT and *Kat2b*$^{-/-}$ mice 24 hours after sepsis induction (n = 4 mice per group). Data are representative of at least three independent experiments (**A** to **F**) and are shown as means ± SD. Statistics: one-way ANOVA. $^{*}P < 0.05$, $^{**}P < 0.01$, $^{***}P < 0.001$, $^{****}P < 0.0001$.

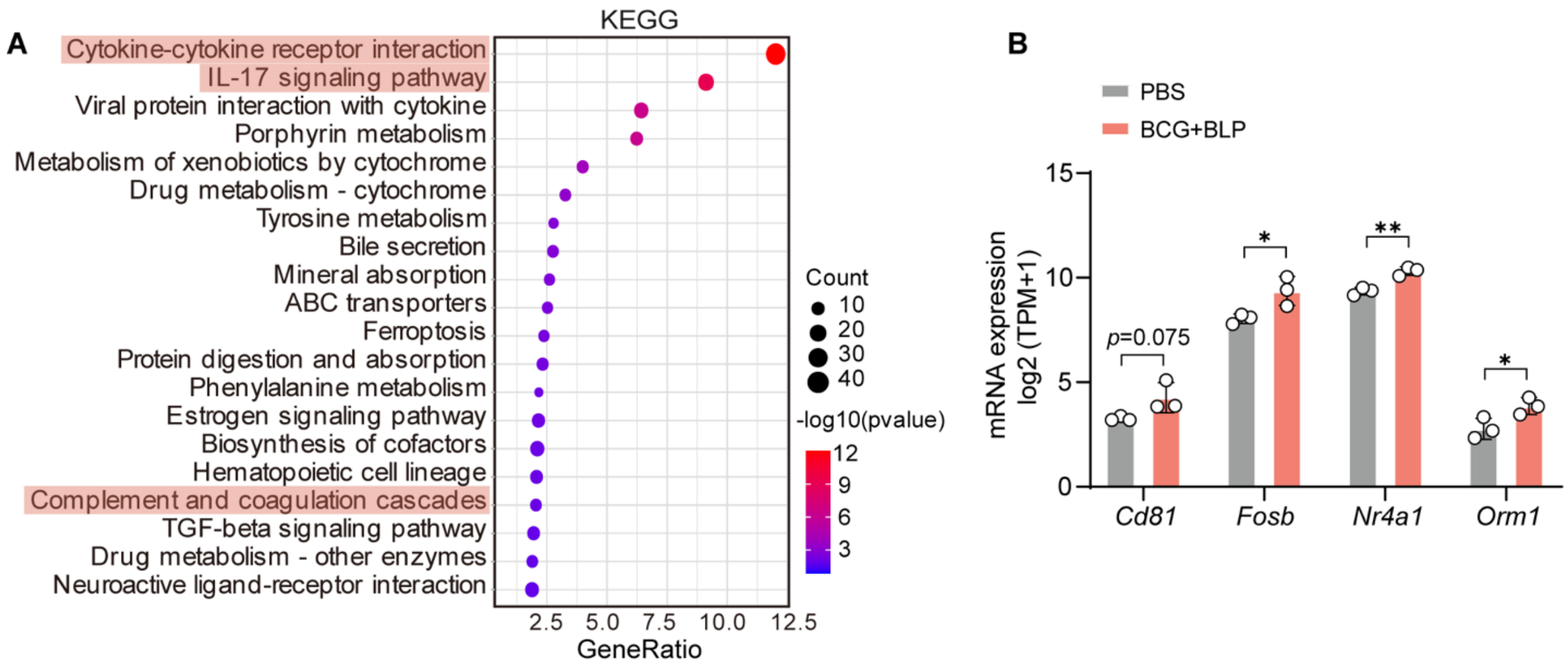

Fig. S7. H3K18 lactylation promotes transcription of phagocytic and inflammatory genes in CCR5^{hi} monocytes, related to Fig. 7. (**A**) KEGG pathway enrichment analysis of upregulated genes in BCG+BLP-trained group based on RNA-seq. (**B**) Comparison of mRNA expression levels of *Cd81*, *Fosb*, *Nr4a1*, and *Orm1* between PBS and BCG+BLP-trained groups in CCR5^{hi} monocytes. Expression levels are shown as log2(TPM+1) based on RNA-seq data. Data are shown as means ± SD. Statistics: Student's *t* test (**B**). $*P < 0.05$, $**P < 0.01$, $***P < 0.001$, $****P < 0.0001$.

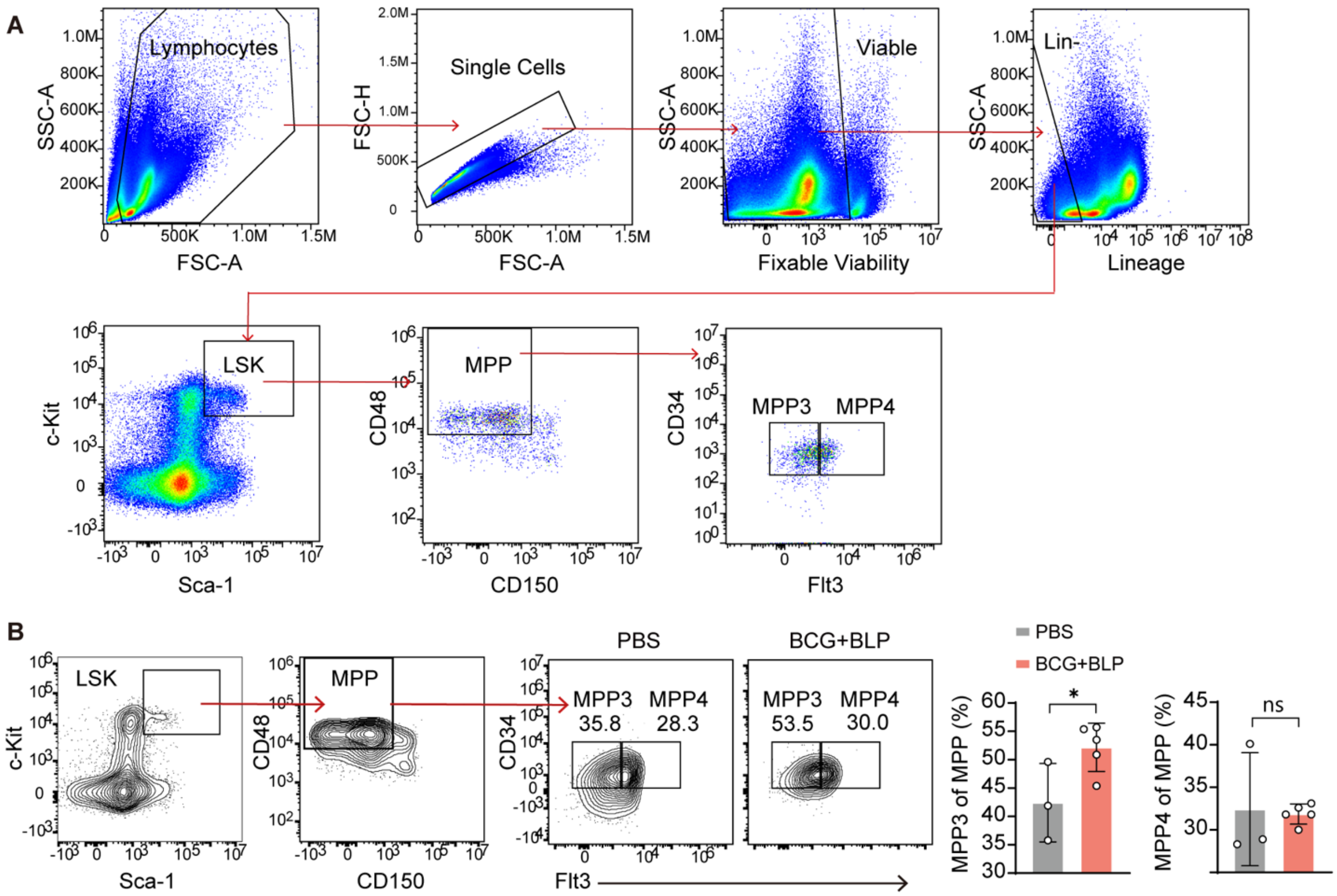

Fig. S8. BCG+BLP induces myeloid-biased hematopoietic reprogramming in bone marrow progenitors. (**A**) Gating strategy for mouse bone marrow HSPCs by flow cytometry. (**B**) Flow cytometric analysis of MPP3s ($CD150^{-}CD48^{+}CD34^{+}Flt3^{-}$ LSKs) and MPP4s ($CD150^{-}CD48^{+}CD34^{+}Flt3^{+}$ LSKs) in the indicated groups, with quantification (n = 3 to 5 mice per group). Data are representative of at least two independent experiments (**B**) and are shown as means ± SD. Statistics: Student's *t* test. ns, not significant; *$P < 0.05$.

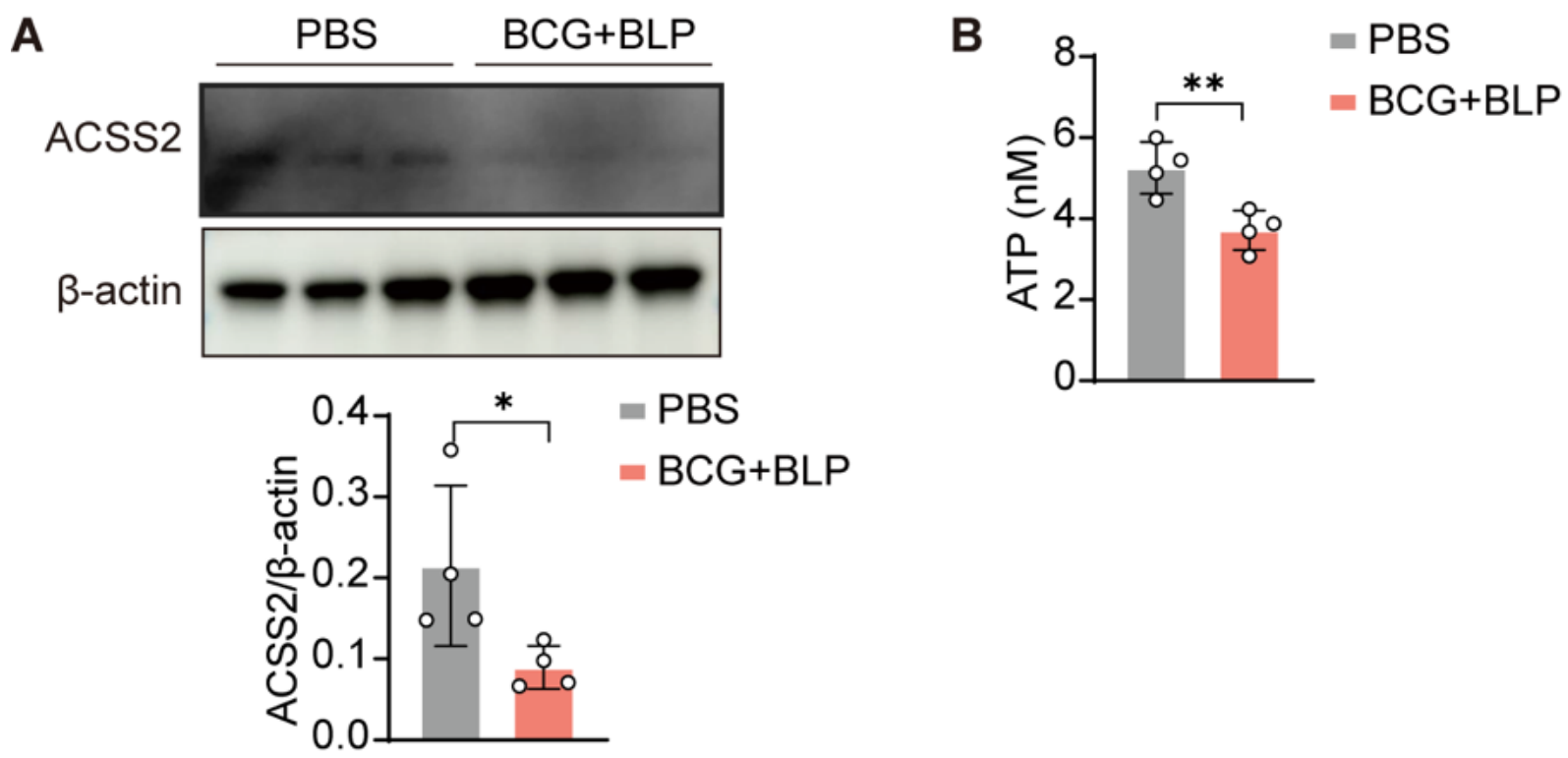

Fig. S9. Assessment of alternative metabolic pathways contributing to H3K18 lactylation in CCR5^{hi} monocytes. (**A**) Western blot analysis of ACSS2 levels in CCR5^{hi} monocytes from the indicated groups, with actin as loading control. Representative immunoblots and densitometry are shown (n = 4). (**B**) Intracellular adenosine 5'-triphosphate (ATP) levels in CCR5^{hi} monocytes measured using a luminometer (n = 4). Data are representative of at least three independent experiments (**A** and **B**) and are shown as means ± SD. Statistics: Student's *t* test (**A** and **B**). *$P < 0.05$, **$P < 0.01$.

Table S1. Key resource

REAGENT or RESOURCE	SOURCE	IDENTIFIER
Antibodies		
anti-Pan Kla	PTM BIO	Cat#PTM-1401
anti-H3K9la	PTM BIO	Cat#PTM-1419RM
anti-H3K14la	PTM BIO	Cat#PTM-1414RM
anti-H3K18la	PTM BIO	Cat#PTM-1406RM
anti-H3K18la	PTM BIO	Cat#PTM-1427RM
anti-H4K5la	PTM BIO	Cat#PTM-1409
anti-H4K8la	PTM BIO	Cat#PTM-1415RM
anti-H4K12la	PTM BIO	Cat#PTM-1411RM
anti-H4K16la	PTM BIO	Cat#PTM-1417RM
anti-Histone H3	Abcam	Cat#ab1791
anti-Histone H4	Abcam	Cat#ab177840
anti-Ly-6C-APC (clone AL-21)	BD Biosciences	Cat#560595
anti-Ly-6G-BV421 (clone 1A8)	BD Biosciences	Cat#562737
PE anti-mouse/human CD11b	Biolegend	Cat#101208
PE/Cyanine7 anti-mouse CD195 (CCR5)	Biolegend	Cat#107018
Brilliant Violet 421 anti-human CD195 (CCR5)	Biolegend	Cat#359118
PerCP/Cyanine5.5 anti-mouse CD45	Biolegend	Cat#103132
PE anti-human CD45	Biolegend	Cat#304007
APC anti-human CD14	Biolegend	Cat#325608
FITC anti-human CD16	Biolegend	Cat#302006
anti-p300	Cell Signaling Technology	Cat#70088
anti-KAT2B	Cell Signaling Technology	Cat#3378
anti-mTOR (7C10)	Cell Signaling Technology	Cat#2983
anti-phospho-mTOR (Ser2448)	Cell Signaling Technology	Cat#2971
anti-Akt(C67E7)	Cell Signaling Technology	Cat#4691
anti-phospho-Akt (Ser473)	Cell Signaling Technology	Cat#4060
anti-HIF-1α	Wuhan Sanying Biotechnology	Cat#20960-1-AP
anti-β-Actin antibody	Wuhan Sanying Biotechnology	Cat#66009-1-Ig
Goat anti-Mouse IgG (H + L) Secondary Antibody, HRP	Jackson ImmunoResearch	Cat#115-035-003
Chemicals, Peptides, and Recombinant Proteins		
Maraviroc	Selleck	Cat#S2003

Syrosingopine (SU 3118)	Selleck	Cat#S9907
2-Deoxy-D-glucose	TargetMol	Cat#T6742
Sodium oxamate	TargetMol	Cat#T19831
(L)-Sodium lactate (NaLa)	TargetMol	Cat#T4103
Lipopolysaccharide from Escherichia coli	Sigma-Aldrich	Cat#L2880
XF Cell Mito Stress Test Kit	Agilent Technologies	Cat#103015-100
XF Glycolysis Stress Test Kit	Agilent Technologies	Cat#103020-100
XFe24 FluxPak	Agilent Technologies	Cat#102340-100
XF DMEM Base Medium	Agilent Technologies	Cat#103575-100
XF 1.0 M Glucose Solution	Agilent Technologies	Cat#103577-100
XF 100 mM Pyruvate Solution	Agilent Technologies	Cat#103578-100
XF 200 mM Glutamine Solution	Agilent Technologies	Cat#103579-100
pHrodo Green BioParticles Phagocytosis Kit	Invitrogen	Cat#P35381
Bacterial Strains		
BCG	Chengdu Institute of Biological Products Co. Ltd (Chengdu, China)	N/A
E. coli	ATCC	Cat#25922

Table S2. Clinical characteristics of enrolled mothers and cord blood samples

No.	Maternal Age (years)	Gestational Age	Gravidity / Parity	Delivery Mode	Pregnancy Complications	Maternal BCG Vaccination History	Vaccination During Pregnancy
1	37	38 weeks	G3P1	VD	None	Yes	None
2	29	38+6 weeks	G3P3	VD	None	Yes	None
3	26	37 weeks	G1P1	VD	Gestational diabetes mellitus	Yes	None
4	28	38+1 weeks	G2P2	VD	Gestational hypertension; anemia	Yes	None
5	32	39+6 weeks	G1P1	VD	None	Yes	None
6	35	40+2 weeks	G2P2	VD	Hypothyroidism	Yes	None
7	35	39+3 weeks	G3P2	VD	Gestational diabetes mellitus	Yes	None
8	29	39 weeks	G1P1	VD	None	Yes	None
9	21	39+4 weeks	G2P1	VD	Hypothyroidism	Yes	None

VD: Vaginal delivery

Data S1. Full, uncropped Western blots.

Fig. 5H

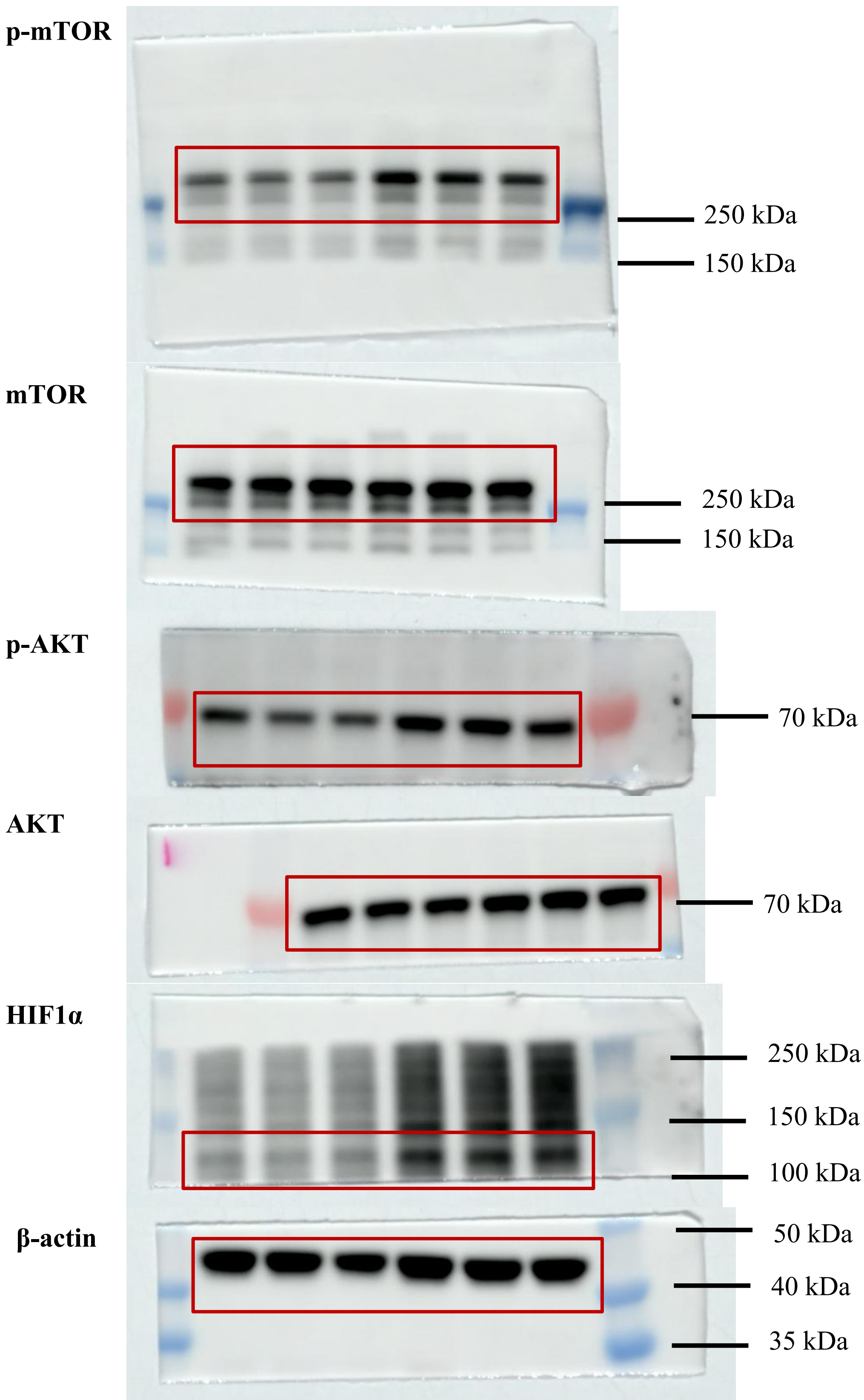
p-mTOR
250 kDa
150 kDa
mTOR
250 kDa
150 kDa
p-AKT
70 kDa
AKT
70 kDa
HIF1α
250 kDa
150 kDa
100 kDa
β-actin
50 kDa
40 kDa
35 kDa

Fig. 6A

Pan-Kla

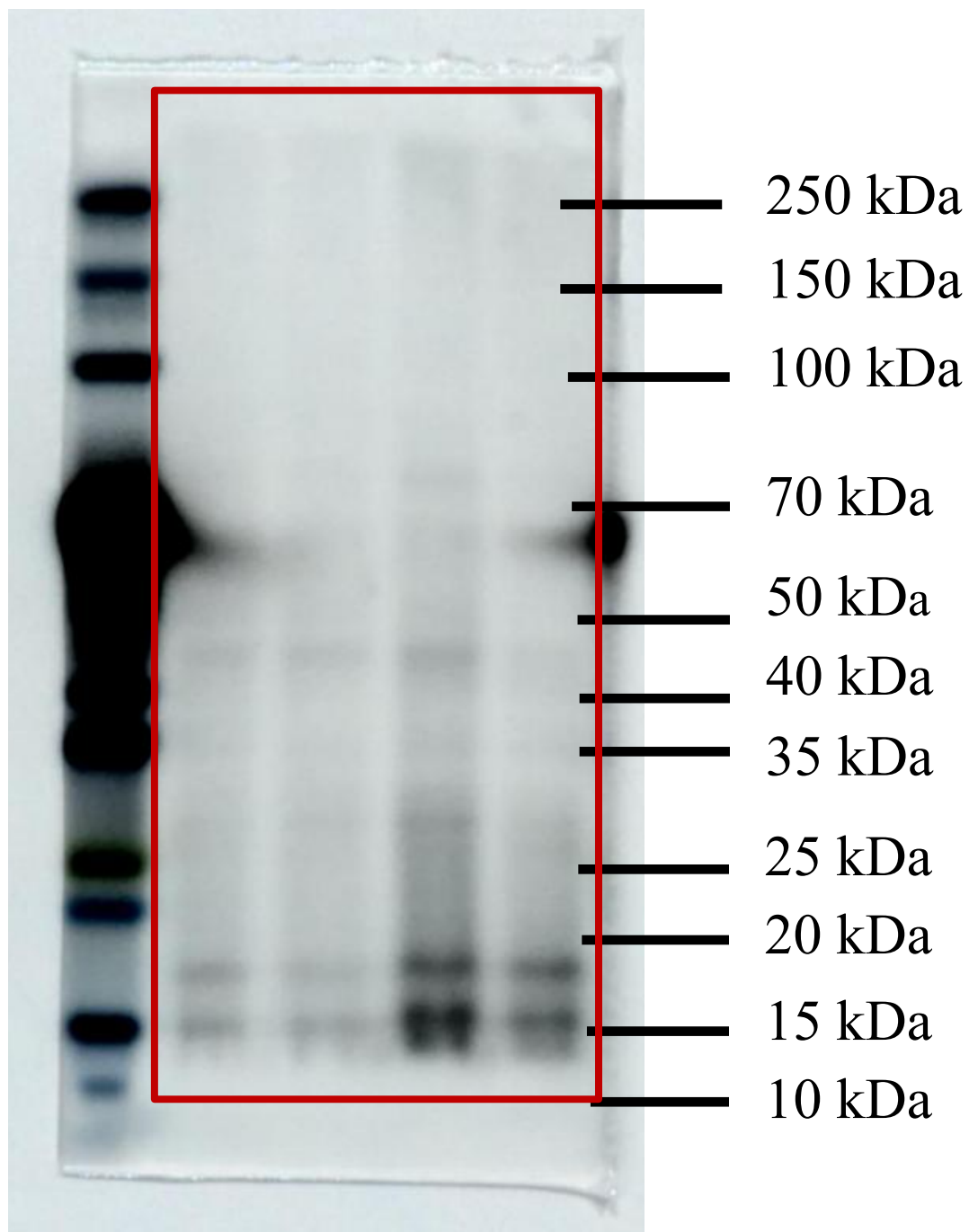

β-actin

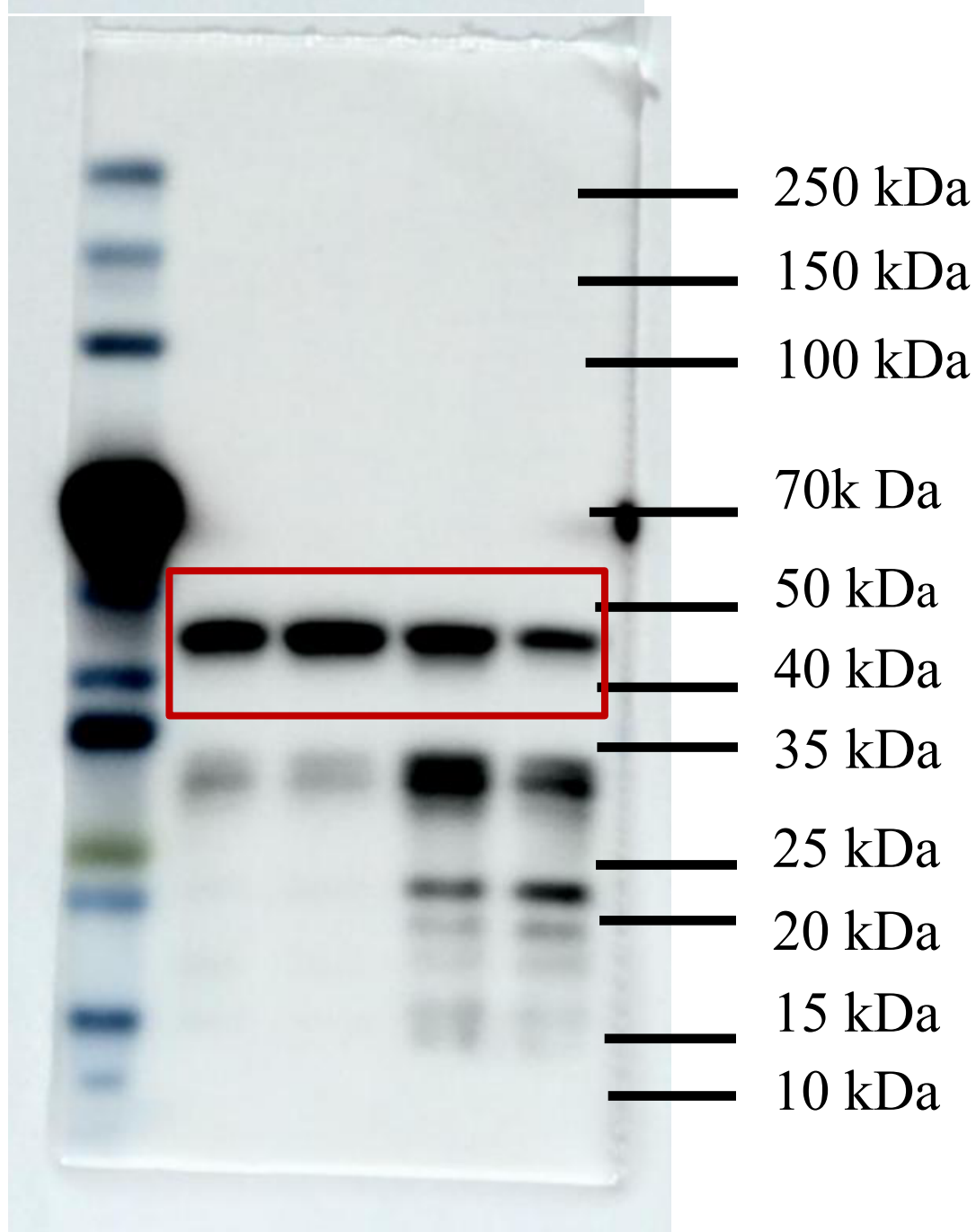

Fig. 6B

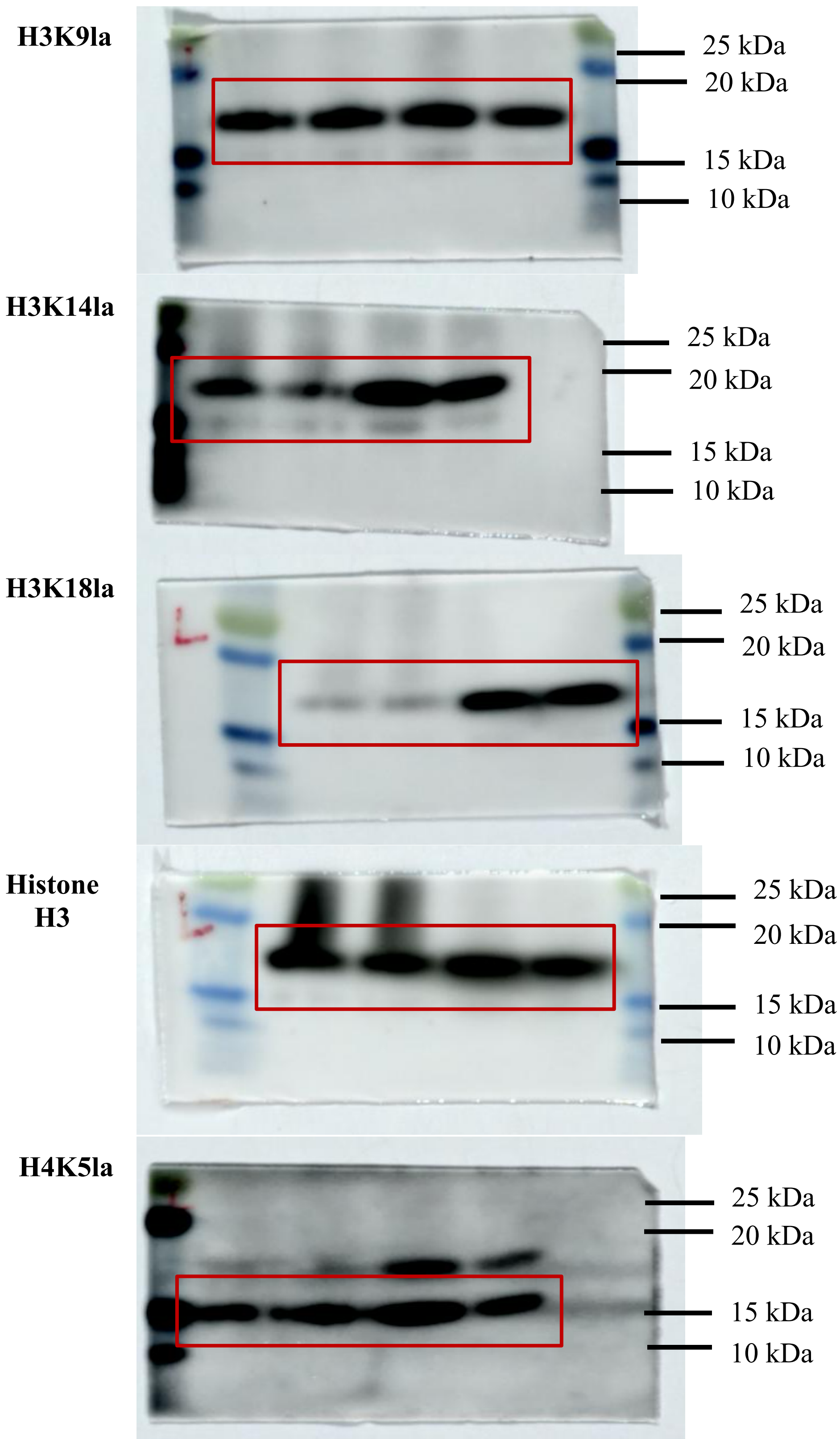
H3K9la
25 kDa
20 kDa
15 kDa
10 kDa
H3K14la
25 kDa
20 kDa
15 kDa
10 kDa
H3K18la
25 kDa
20 kDa
15 kDa
10 kDa
Histone H3
25 kDa
20 kDa
15 kDa
10 kDa
H4K5la
25 kDa
20 kDa
15 kDa
10 kDa

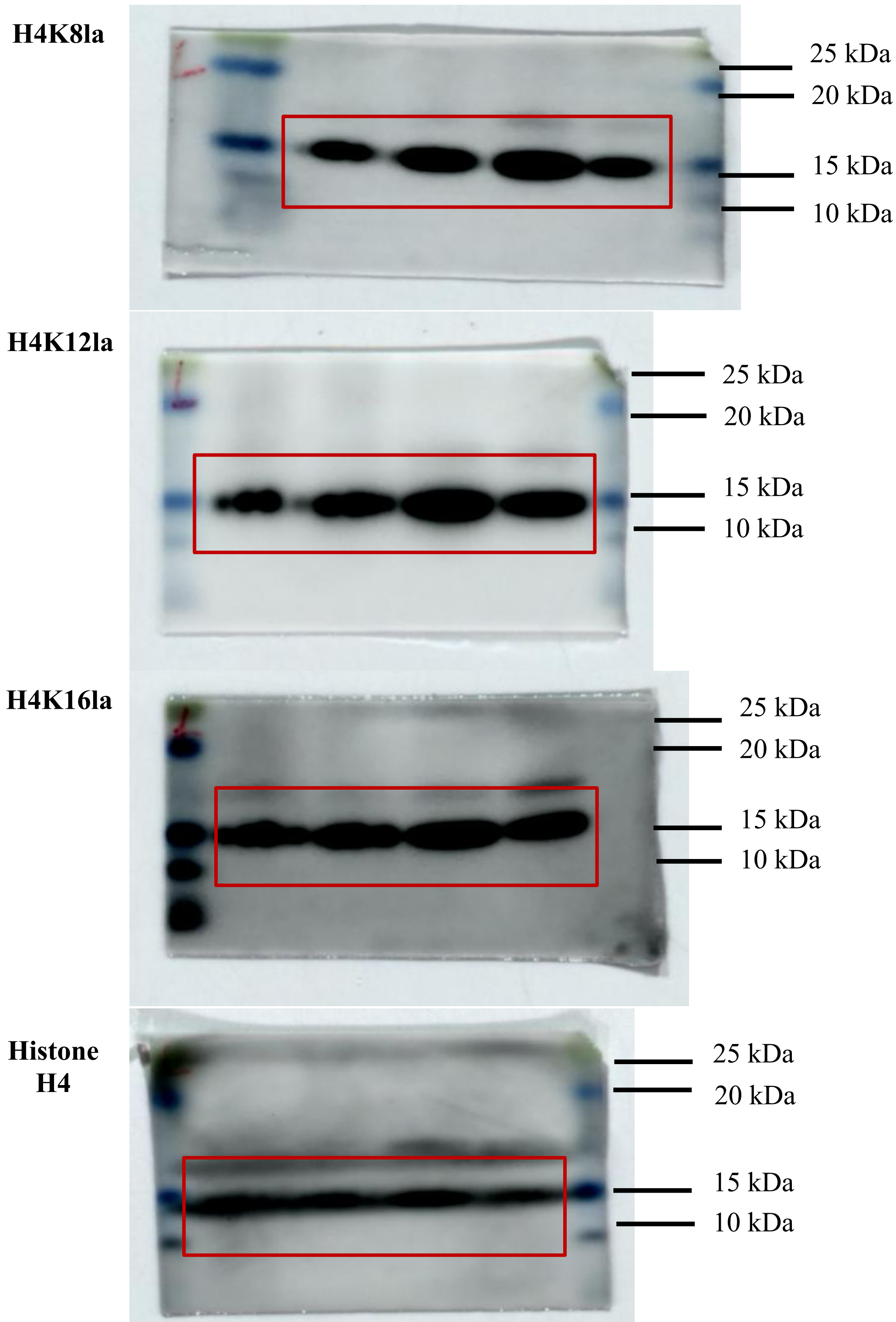
H4K8la
25 kDa
20 kDa
15 kDa
10 kDa
H4K12la
25 kDa
20 kDa
15 kDa
10 kDa
H4K16la
25 kDa
20 kDa
15 kDa
10 kDa
Histone H4
25 kDa
20 kDa
15 kDa
10 kDa

Fig. 6C

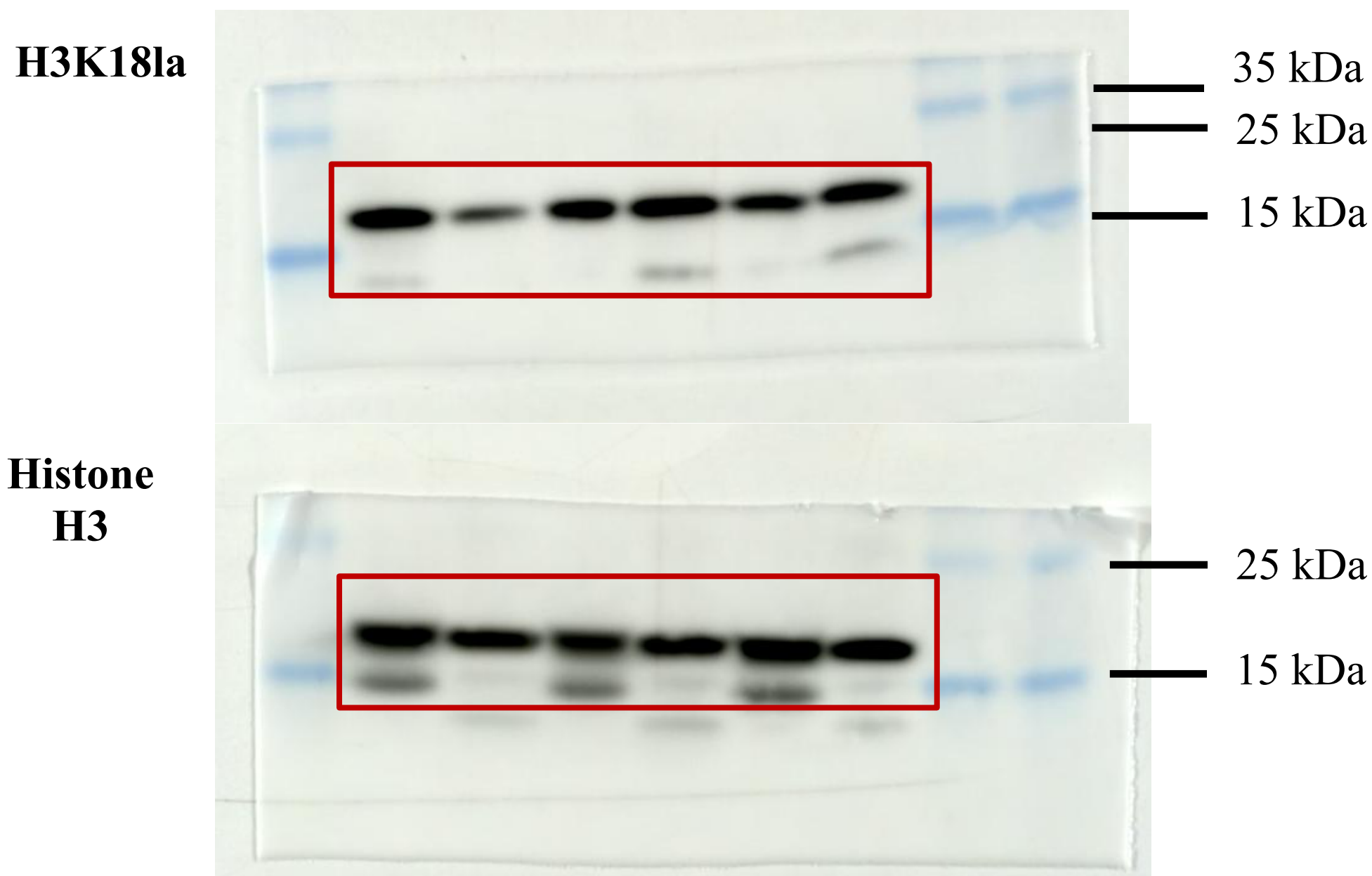
H3K18la
35 kDa
25 kDa
15 kDa
Histone H3
25 kDa
15 kDa

Fig. 6E

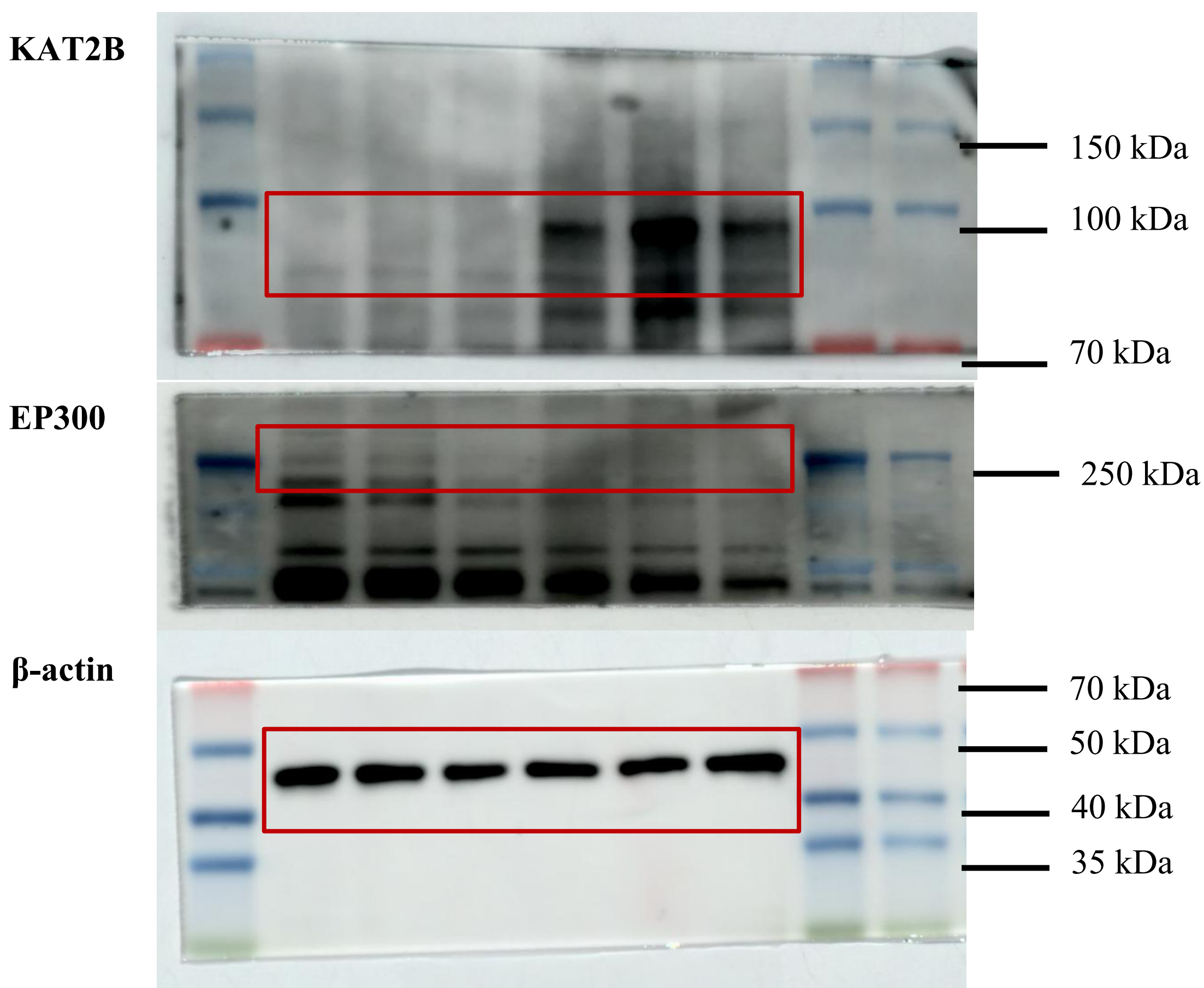
KAT2B
150 kDa
100 kDa
70 kDa
EP300
250 kDa
β-actin
70 kDa
50 kDa
40 kDa
35 kDa

Fig. 6G

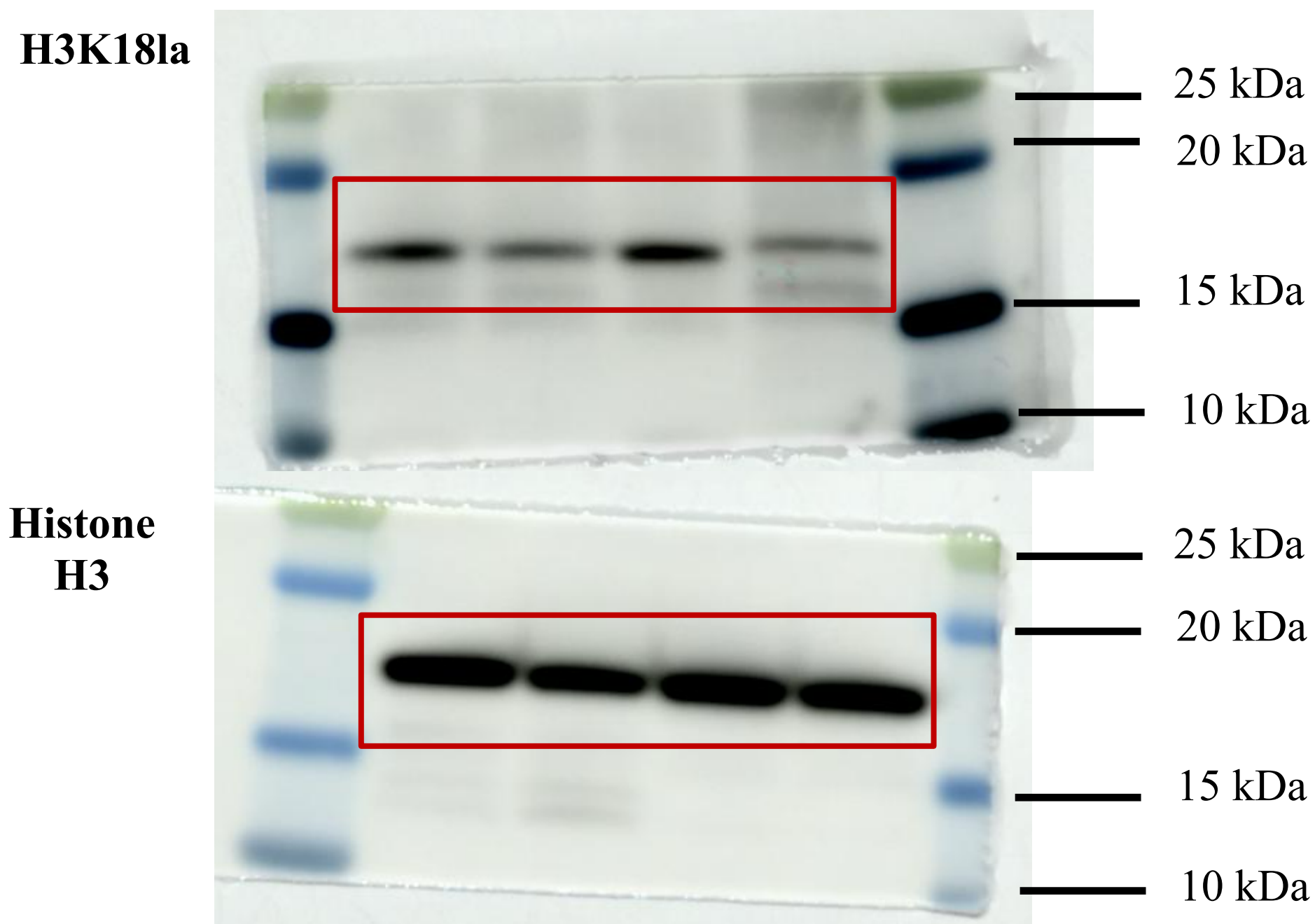
H3K18la
25 kDa
20 kDa
15 kDa
10 kDa
Histone H3
25 kDa
20 kDa
15 kDa
10 kDa

Fig. 6J

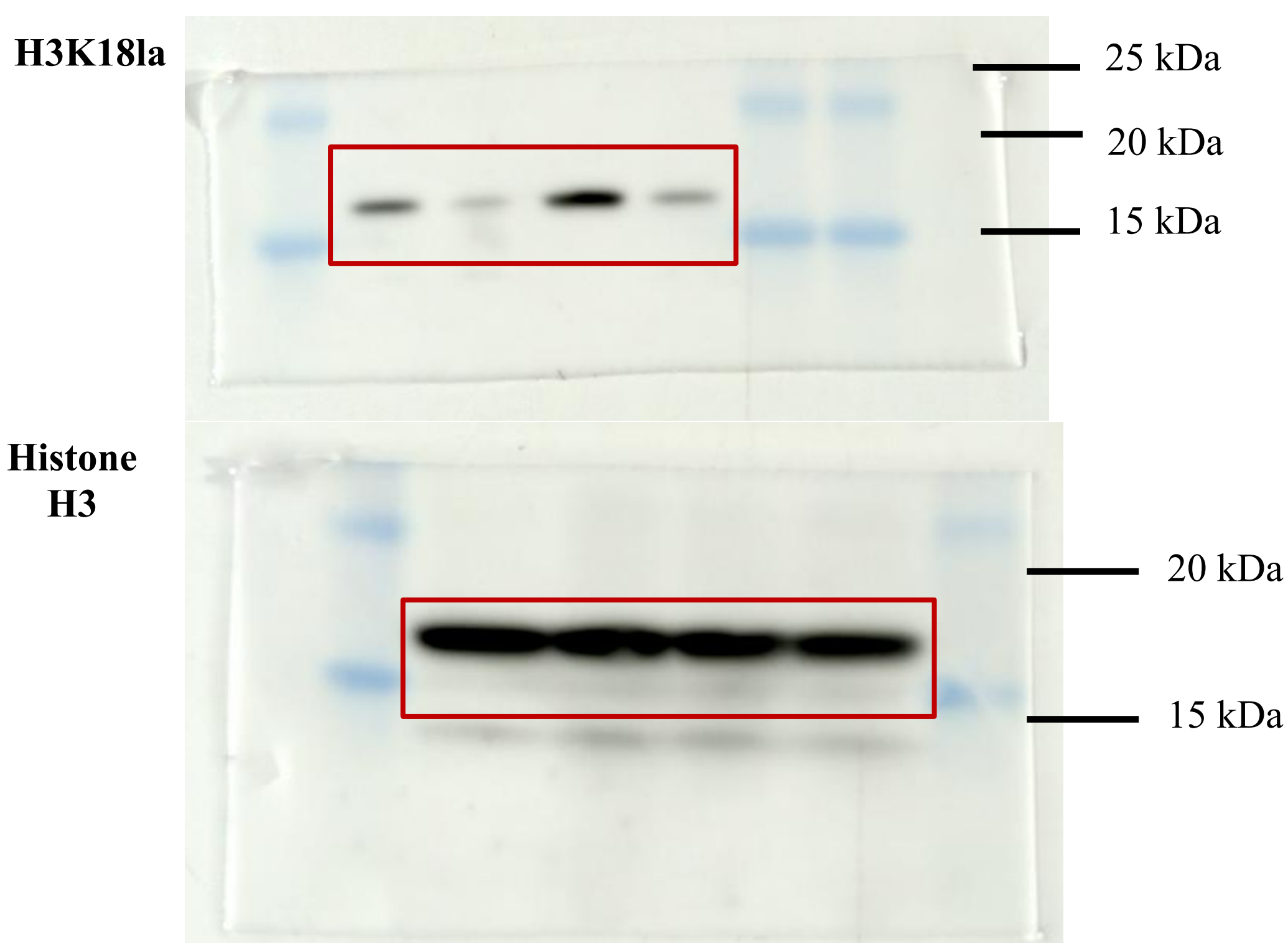
H3K18la
25 kDa
20 kDa
15 kDa
Histone H3
20 kDa
15 kDa

Fig. 8E

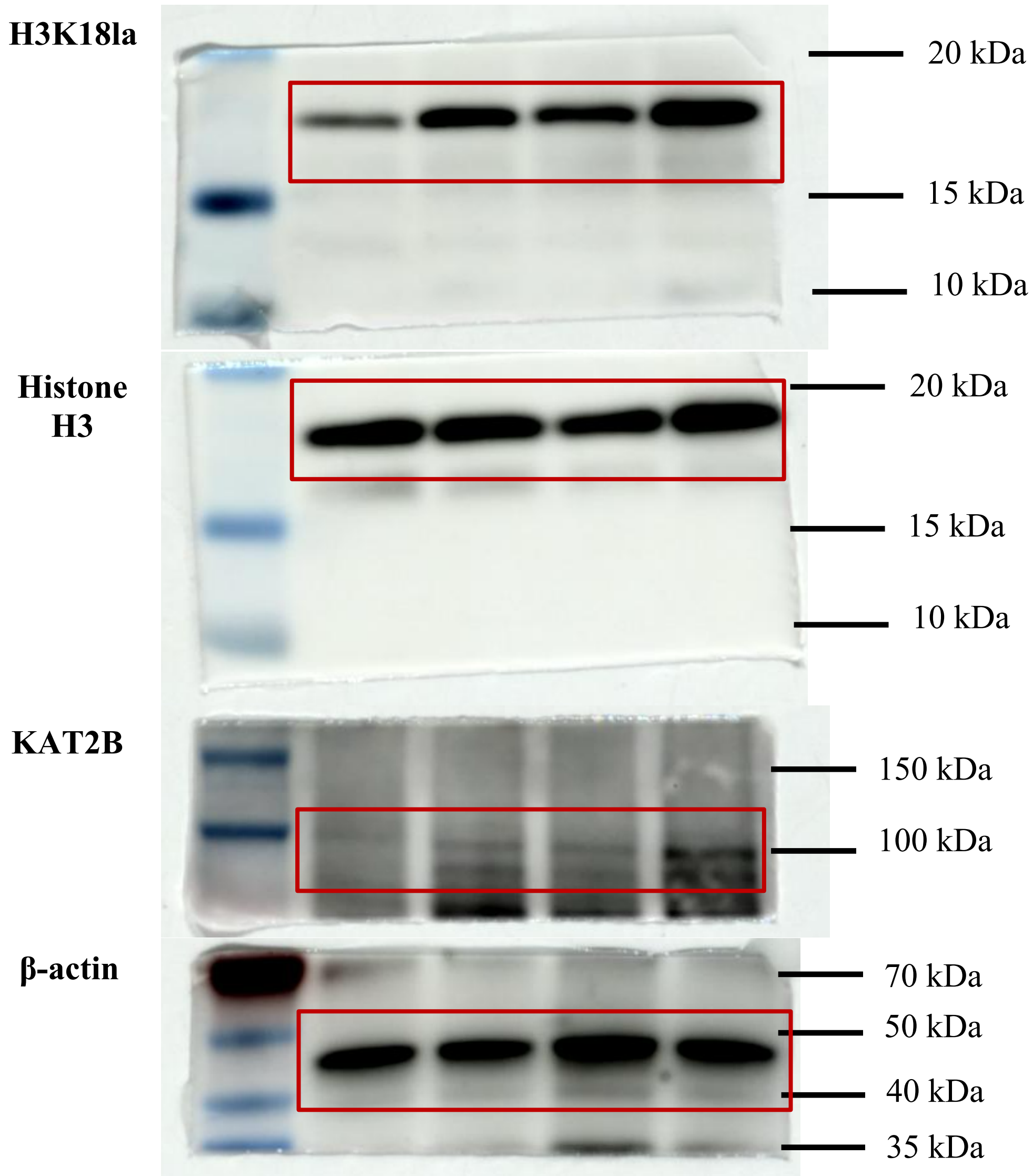

H3K18la
20 kDa
15 kDa
10 kDa
Histone H3
20 kDa
15 kDa
10 kDa
KAT2B
150 kDa
100 kDa
β-actin
70 kDa
50 kDa
40 kDa
35 kDa

Fig. S6A

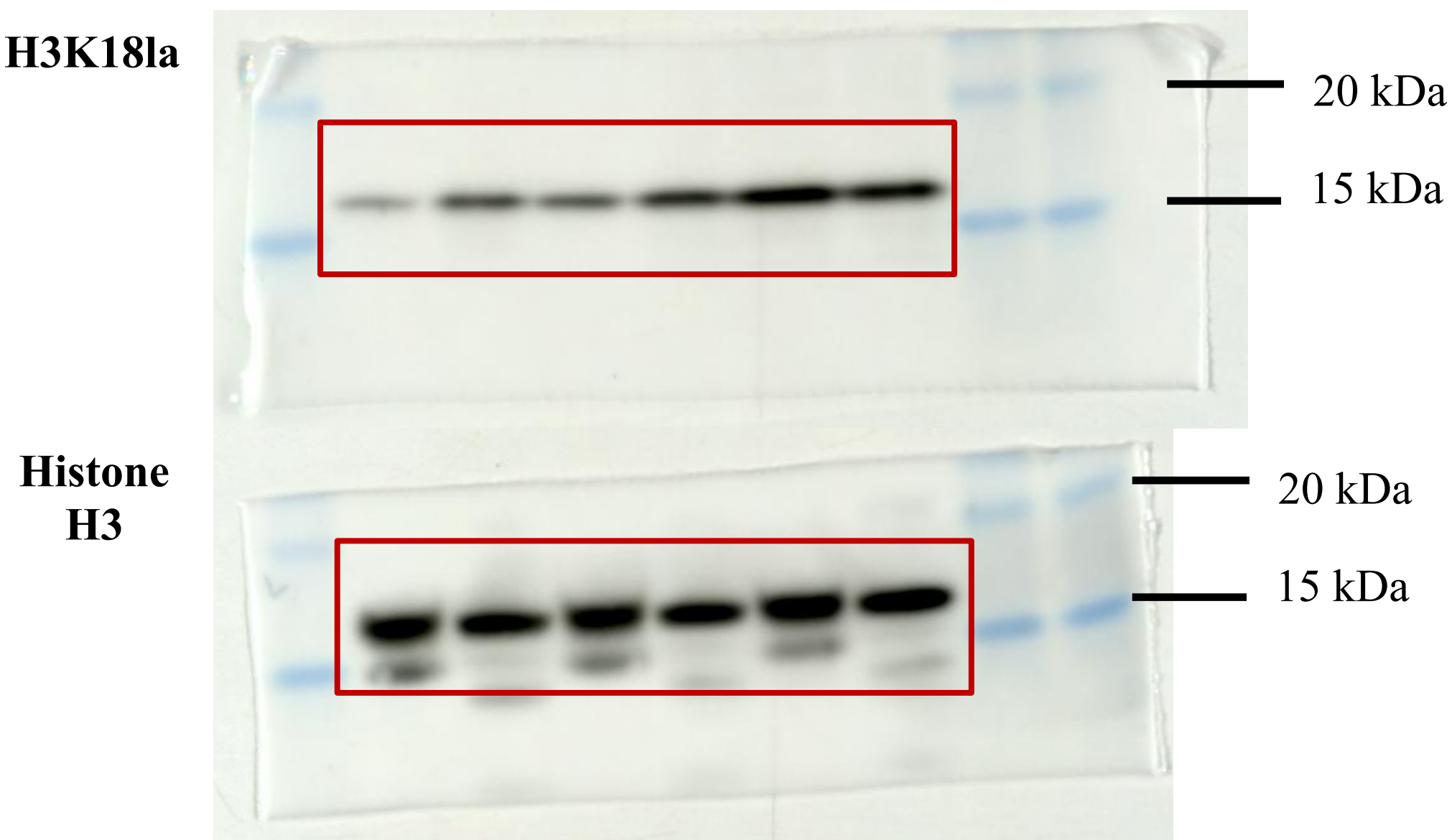

Fig. S6B

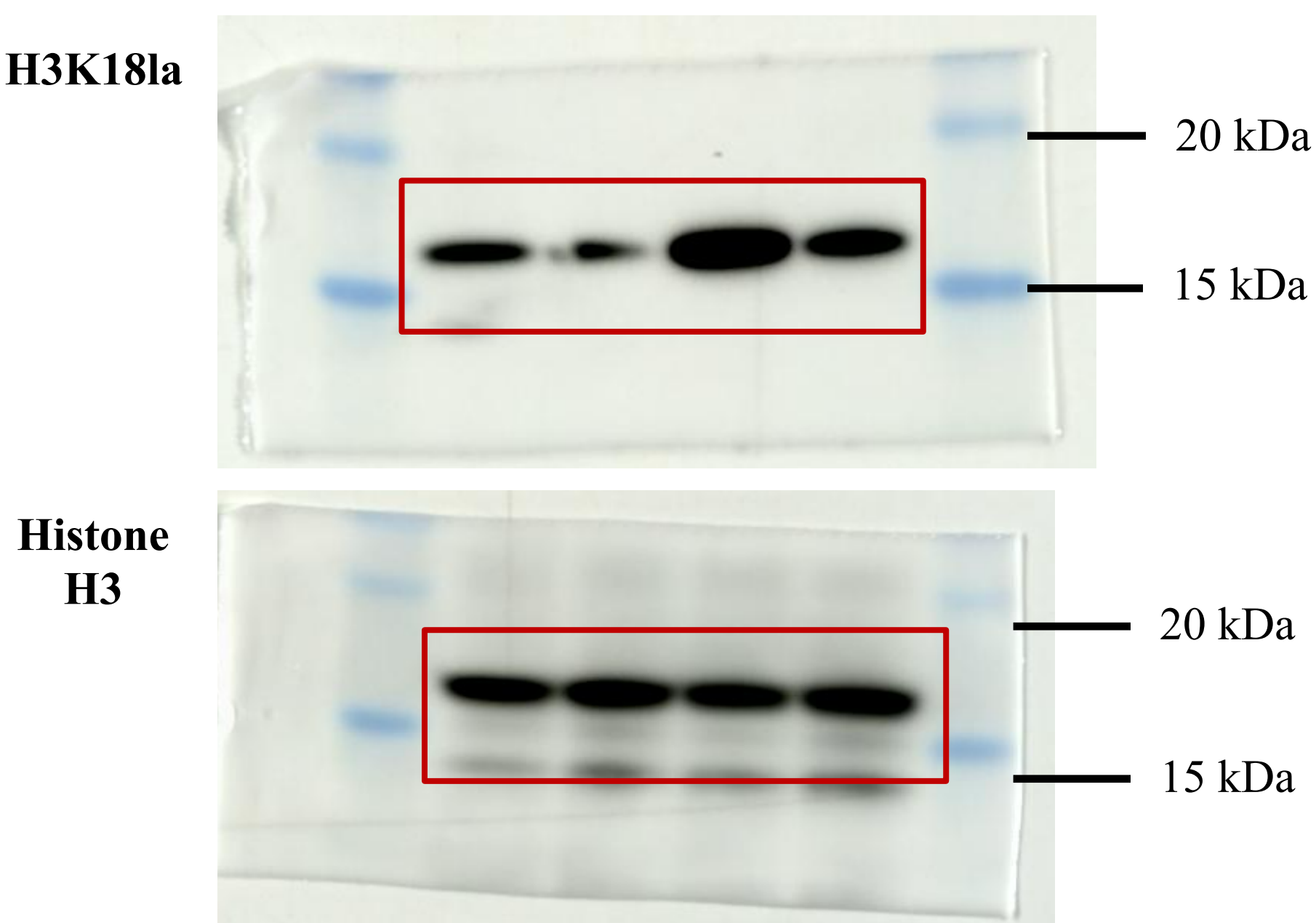

Fig. S6C

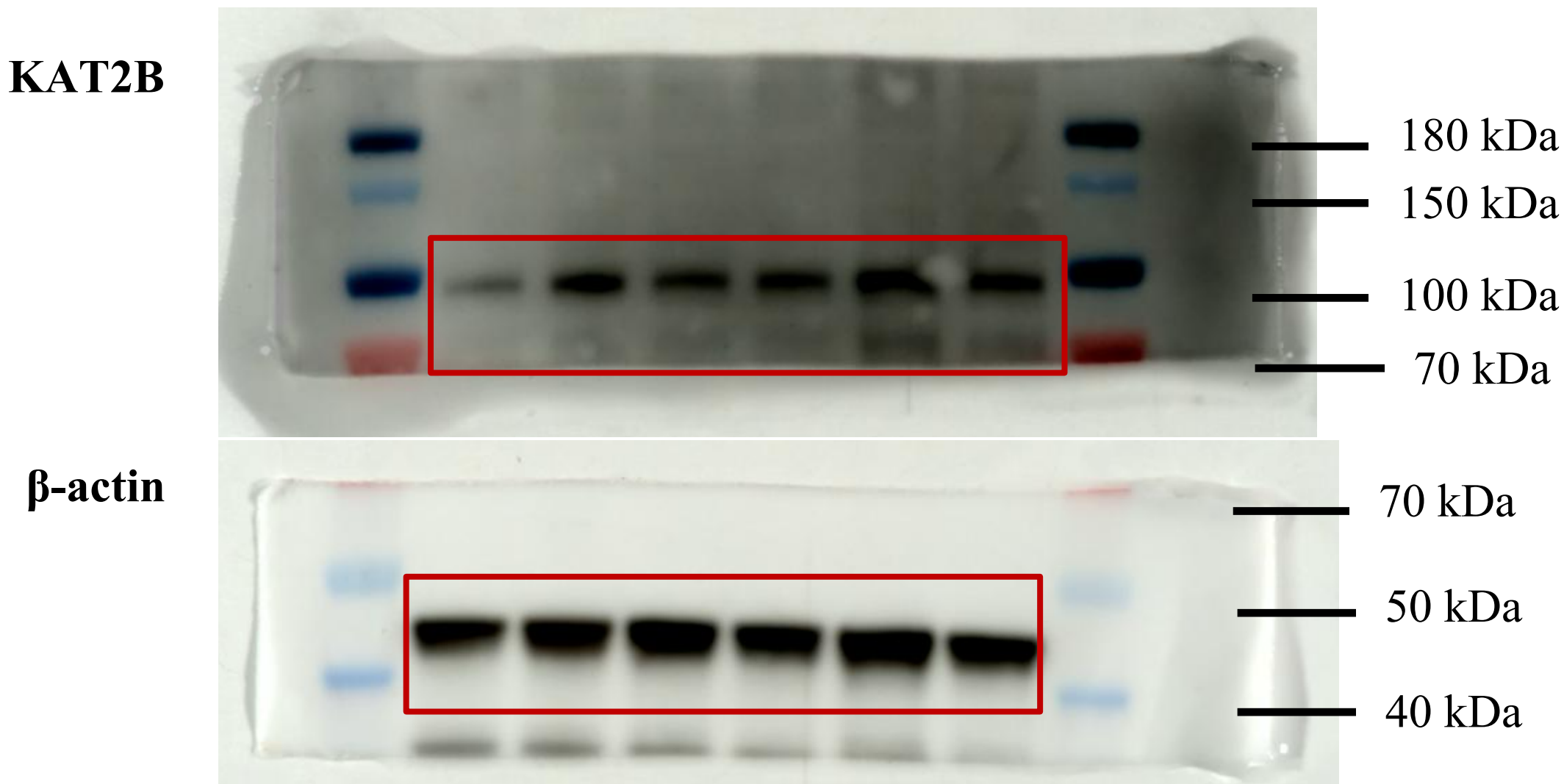

Supplement: Supplementary file 1 [file sciadv.aee1634_sm.pdf]
